# Duck plague virus LORF2 utilizes RNF34 to inhibit antiviral innate immunity by ubiquitination and degradation of IRF7

**DOI:** 10.1371/journal.ppat.1014174

**Published:** 2026-04-24

**Authors:** Yanming Tian, Bin Tian, Ran Ran, Dongjie Cai, Zhen Xiao, Mingshu Wang, Ying Wu, Qiao Yang, Shaqiu Zhang, Dekang Zhu, Mafeng Liu, Xinxin Zhao, Di Sun, Juan Huang, Xumin Ou, Zhen Wu, Yu He, Renyong Jia, Shun Chen, Anchun Cheng

**Affiliations:** 1 Engineering Research Center of Southwest Animal Disease Prevention and Control Technology for Ministry of Education of the People’s Republic of China, International Joint Research Center for Animal Disease Prevention and Control of Sichuan Province, Key Laboratory of Animal Disease and Human Health of Sichuan Province, Research Center of Avian Disease and Institute of Veterinary Medicine and Immunology, College of Veterinary Medicine, Sichuan Agricultural University, Chengdu, China; 2 Research Center of Avian Disease, College of Veterinary Medicine, Sichuan Agricultural University, Chengdu, Sichuan, China; 3 Agricultural Animal Diseases and Veterinary Public Health Key Laboratory of Sichuan Province, Sichuan Agricultural University, Chengdu, Sichuan, China; 4 Institute of Veterinary Immunology and Green Drugs, Veterinary Department in College of Animal Science, State Key Laboratory of Green Pesticide, Guizhou University, Guiyang, China; University of Illinois at Chicago, UNITED STATES OF AMERICA

## Abstract

Duck plague, caused by the alphaherpesvirus Duck plague virus (DPV), is an acute, hemorrhagic, and economically devastating disease of waterfowl. DPV infection induces severe immunosuppression, yet the mechanisms by which this pathogen subverts host innate immunity, particularly through manipulation of the host ubiquitin system, remain unclear. The cGAS-STING signaling pathway is a cornerstone of anti-DNA viral immunity. In avian species, where IRF3 has been evolutionarily lost, the transcription factor IRF7 plays a pivotal role in activating type I interferons (IFN-I). Here, we identify duck RNF34 (DuRNF34) as a host E3 ubiquitin ligase that broadly suppresses the duck cGAS-STING pathway by targeting multiple components, including DucGAS, DuSTING, and DuIRF7, for ubiquitination and degradation. Importantly, DPV infection upregulates DuRNF34 expression, which selectively targets DuIRF7 for degradation to facilitate viral replication. Further affinity purification-mass spectrometry (AP-MS) analysis revealed that LORF2, a DPV-specific protein, recruits DuRNF34 to catalyze K11- and K48-linked polyubiquitination of DuIRF7 at lysine residues K51 and K453, leading to DuIRF7 degradation and suppression of IFN-β and downstream antiviral genes. Functional validation confirmed that siRNA-mediated knockdown of LORF2 markedly attenuated DPV-induced DuIRF7 degradation and impaired viral replication. Collectively, these findings reveal a novel immune evasion strategy in which DPV hijacks the host E3 ligase DuRNF34 via its unique protein LORF2, thereby targeting DuIRF7 for degradation to subvert innate immunity. This work provides new insights into herpesviral immune evasion and suggests potential targets for therapeutic intervention.

## Author summary

Duck plague is a severe disease in waterfowl caused by Duck plague virus (DPV), which is a highly pathogenic avian herpesvirus and must overcome the host’s innate immunity to cause disease. The cGAS-STING signaling pathway is a crucial defense mechanism of the host, and birds rely entirely on the transcription factor IRF7 to activate antiviral interferons. This study reveals a new strategy by which DPV undermines this key host defense. We found that the host protein DuRNF34, an E3 ubiquitin ligase, naturally inhibits the cGAS-STING pathway. DPV infection not only increased the level of DuRNF34, but also hijacked this host enzyme through its unique viral protein LORF2. LORF2, as a molecular adapter, recruits DuRNF34 to specifically mark IRF7 for degradation, thereby shutting down the interferon response and promoting viral replication. This work uncovers a precise mechanism of viral immune evasion, highlights the therapeutic potential of targeting the LORF2-DuRNF34 interaction, and advances our understanding of how herpesviruses manipulate the host’s ubiquitin system to establish infection.

## Introduction

Duck plague (DP), also referred to as duck viral enteritis (DVE), is an acute, contagious, and septicemic disease caused by Duck plague virus (DPV) [1]. DPV exhibits a host range primarily confined to birds within the order Anseriformes. In addition to ducks, natural and experimental infections have been documented in a variety of waterfowl species, including swans, geese, Canada geese, and muscovy ducks [2–5]. To date, there is no evidence that DPV can cause infection in other bird orders, fish, mammals, or humans [6]. DPV is a double-stranded DNA virus belonging to the *Alphaherpesvirinae* subfamily. Its genome consists of a unique long (UL) region, a unique short (US) region, and terminal and internal short repeat sequences (TRS and IRS), forming a UL-IRS-US-TRS structure [7]. It is known that herpes simplex virus 1 (HSV-1) utilizes latent infection to evade host immune surveillance [8]. Similarly, DPV possesses the fundamental biological traits required to establishing latent infection. Specifically, the trigeminal ganglion has been identified as a site of latency, from which the virus can reactivate and cause recrudescent disease [9]. Ducks surviving acute infection can become long-term carriers, shedding virus intermittently for up to four years [10]. Consistent with this neurotropism, our prior work has shown that DPV can infect neuronal cell types, including neurons and astrocytes, in vitro [11]. Among herpesviruses, avian herpesviruses—including DPV, Marek’s disease virus (MDV), infectious laryngotracheitis virus (ILTV), and turkey herpesvirus (HVT)—infect only birds but not mammals. These viruses possess not only genes homologous to other herpesviruses but also a unique set of genes, such as LORFs and SORFs, which play important roles in viral replication and pathogenicity [12]. For instance, large-fragment deletions in MDV vaccine strains involving SORF1, SORF2, SORF3 suggest that these unique genes contribute to viral virulence [13]. Furthermore, studies on MDV have demonstrated that the deletion of LORF2 (also known as vLIP) reduces pathogenicity and improves host survival, whereas the deletion of LORF4 unexpectedly enhances virulence and tumor development [14–16]. Our prior work has revealed the specific roles of unique DPV genes, including LORF3, LORF4, and LORF5, in viral replication and cell-to-cell spread [17–19]. To evade host immunity, DPV employs multiple viral proteins to counteract the cGAS-STING-mediated antiviral innate immune pathway. For instance, UL41 selectively downregulates IRF7 expression [20], US3 targets both STING and IRF7 for phosphorylation-dependent degradation [21,22], and UL46 disrupts the assembly of the STING-TBK1-IRF7 signaling complex [23]. However, whether the DPV-specific LORF2 protein is involved in the regulation of innate immunity and viral replication has not yet been elucidated.

Innate immune response is the main host defense mechanism against microbial invasion [24]. Following viral infection, host pattern recognition receptors (PRRs)—such as RIG-I-like receptors (RLRs), Toll-like receptors (TLRs), and the DNA sensor cyclic GMP-AMP synthase (cGAS)—specifically recognize viral pathogen-associated molecular patterns (PAMPs), including viral nucleic acids, proteins, and carbohydrates, this recognition initiates the innate immune response [25,26]. Among these pathways, the cGAS-STING signaling axis plays a central role in detecting cytosolic viral DNA and triggering the production of type I interferons (IFN-I). Briefly, after binding viral DNA, cGAS synthesizes the second messenger 2’3’-cyclic GMP-AMP (cGAMP) using ATP and GTP. cGAMP then activates stimulator of interferon genes (STING), which translocates from the endoplasmic reticulum (ER) to the Golgi apparatus. During this process, STING recruits and activates TANK-binding kinase 1 (TBK1) and interferon regulatory factors 3 and 7 (IRF3/7), ultimately leading to IFN-I secretion [27,28]. Given the critical role of this pathway in antiviral immunity, many viruses have evolved strategies to suppress it. For example, HSV-1 protein VP22 inhibits cGAS enzymatic activity [29], porcine reproductive and respiratory syndrome virus (PRRSV) Nsp5 impedes STING translocation and activation, and the African swine fever virus (ASFV) E120R protein blocks TBK1-IRF3 complex formation, thereby suppressing IRF3 activation [30]. Notably, avian species have lost the IRF3 ortholog through evolution, rendering IRF7 essential for signal transduction via the cGAS-STING pathway and for IFN expression during antiviral responses [31]. Consistent with this critical role, duck has been shown to restrict the dissemination of Newcastle disease virus (NDV) in cells by amplifying the transcription of immune related factors [32]. Similarly, goose IRF7 promotes the expression of IFN and pro-inflammatory cytokines, thereby inhibiting the replication of both NDV and vesicular stomatitis virus (VSV) [33]. However, the mechanisms by which DNA viruses target and regulate IRF7 in avian remain to be elucidated.

Ubiquitination is a three-step enzymatic process involving a ubiquitin-activating enzyme (E1), a ubiquitin-conjugating enzyme (E2), and a ubiquitin ligase (E3). The E3 ligase provides substrate specificity and determines the outcome of the modification, making it essential for the specificity of the ubiquitination system [34]. Based on their structural domains, E3 ubiquitin ligases are classified into four major families: RING, U-box, Cullin-RING, and HECT types [35]. As the key mediators of ubiquitination, E3 ubiquitin ligases coordinate diverse cellular processes. Accumulating evidence highlights their crucial role in regulating innate immunity and viral infection. For instance, duck TRIM13 modulates type I interferon production by targeting MAVS for SQSTM1-mediated selective autophagy [36], while Triad3A negatively regulates the RIG-I RNA sensing pathway by mediating Lys48-linked ubiquitination and degradation of the adaptor protein TRAF3 [37]. Viruses can exploit these mechanisms to enhance their infection. For instance, Duck Tembusu virus (DTMUV) suppresses interferon production via the JOSD1-SOCS1-IRF7 negative feedback loop [38], and its NS2B protein recruits the E3 ligase duMARCH5 to promote polyubiquitination of duMAVS, thereby inhibiting MAVS-mediated innate immunity and promoting viral replication [39]. Similarly, PRRSV nonstructural protein 2 enhances Lys63-linked polyubiquitination of RIG-I, leading to autophagic degradation of the adaptor SH3KBP1 and subsequent suppression of host immunity [40]. However, current research has primarily focused on the relationship between E3 ligases and the RLR pathway during RNA virus infection. Their roles within the cGAS-STING pathway in the context of DNA virus infection remain largely unexplored. Numerous studies over the past decades have shown that RNF family E3 ubiquitin ligases are widely involved in the pathogenesis of various diseases, including cancer, immune disorders, viral infections, and neuropsychiatric conditions, underscoring their significant biological relevance. For example, RNF216 suppresses H5N1 avian influenza virus replication and modulates the RIG-I signaling pathway in ducks [41], while RNF123 mediates the ubiquitination and degradation of SOCS1 to regulate type I interferon production during DTMUV infection [42]. Previous studies have indicated that RNF34 is involved in modulating inflammatory responses and RLR-mediated innate immunity [43,44]. However, its potential function within the cGAS-STING signaling pathway, particularly in the context of avian herpesvirus infection, remains largely unexplored.

In this study, we identified DuRNF34 as a negative regulator of the duck cGAS-STING pathway, demonstrating high conservation in avian cells. DuRNF34 interacts with multiple components of this signaling axis, promoting their ubiquitination and subsequent degradation, including DucGAS, DuSTING, and the key transcription factor DuIRF7. Functionally, overexpression of DuRNF34 enhanced DPV proliferation in DEF cells, while DuRNF34 knockdown suppressed viral replication by attenuating DPV induced DuIRF7 degradation. Mechanistically, the DPV unique protein LORF2 recruits DuRNF34 to target DuIRF7 for ubiquitination and degradation, thereby effectively suppressing IFN-I signaling and promoting viral replication. Our findings uncover a novel immune evasion strategy wherein DPV commandeers the host ubiquitin system via a viral-specific factor to dismantle a central antiviral signaling node.

## Materials and methods

### Ethics statement

All animal procedures were performed in strict compliance with institutional guidelines and regulations, and were approved by the Committee of Experimental Operational Guidelines and Animal Welfare at Sichuan Agricultural University (Ethical Approval No. SYXK 2019–187). 10-day-old duck embryos were sourced from a commercial farm that was both free of duck plague virus (DPV) and had not implemented vaccination against DPV.

### Antibodies and reagents

The antibodies and reagents employed in this study are listed below: Rabbit anti-Flag (20543–1-AP), mouse anti-GAPDH (60004–1-Ig), and rabbit anti-HA (51064–2-AP) were obtained from Proteintech. Rabbit anti-Myc (AE102), rabbit anti-GFP (AE078), and rabbit anti-RNF34 (A8517) were acquired from ABclonal Biotechnology; we have commissioned ABclonal to produce a rabbit polyclonal antibody against duck IRF7 (anti-DuIRF7, lot: WG-0305D, 1:1000). This antibody is prepared based on the synthetic peptide segment within the internal region of the duck IRF7 protein (amino acid sequence: NSGNVS(p)LQLSD-C). Rabbit anti-phospho-TBK1 (bs-3440R) was sourced from Bioss. Rabbit anti-TBK1 (T55145), rabbit anti-IgG (B30011), and mouse anti-GST (M20007) were purchased from Abmart. SureBeads Protein A/G magnetic beads (Bio-Rad, 1614013) were used for immunoprecipitation. Horseradish peroxidase (HRP)-conjugated secondary antibodies (Boster Biological Technology, BA1054 and BA1050) were employed for immunoblotting. Poly(dA:dT) (HY-138646), MG132 (HY-13259, with a purity of 99.0% and a working concentration of 10 μM), Bafilomycin A1 (HY-100558, with a purity of 99.95% and a working concentration of 100 nM), and Z-VAD (HY-164388, with a purity of 99.82% and a working concentration of 20 μM) were supplied by Med Chem Express. All inhibitors were dissolved in dimethyl sulfoxide (DMSO) as recommended by the manufacturer to prepare stock solutions, which were aliquoted and stored at -20°C.

### Cells and transfection

Primary duck embryonic fibroblasts (DEF) cells were isolated from 10-day-old duck embryos following established protocols [11], and primary chicken embryo fibroblasts (CEF) cells were prepared as described above. Duck Embryo Hepatocytes (DEH) cells were isolated from 14-day-old duck embryos as previously reported [45]. DEF cells, CEF cells, DEH cells and human embryonic kidney 293T (HEK293T) cells were cultured in Dulbecco’s Modified Eagle Medium (DMEM; Gibco) containing 10% fetal bovine serum (FBS; Yeasen) and kept at 37 °C in a humidified atmosphere with 5% CO₂. Transfections of plasmids, shRNAs, and siRNAs were performed with Lipofectamine 2000 (Lablead, TR001) in accordance with the manufacturer’s guidelines.

### Plasmids and shRNA/siRNA

Duck cGAS, STING, TBK1, IRF7, and RING-type E3 ubiquitin ligases, along with their truncated mutants, were amplified from DEF cells genomic DNA. Chicken RNF34 were amplified from CEF cells genomic DNA. Chicken cGAS, STING, TBK1 and IRF7 plasmids were gifted by Dr. Li Gao, and Dr. Kai Li (Harbin Veterinary Research Institute, China). The DPV gene was constructed by cloning the cDNA of DPV-CHv. All plasmids were generated using the pCAGGS empty vector as the backbone. Primer sequences for plasmid construction were designed and synthesized by Tsingke Biotechnology (Beijing, China), and all constructed plasmids were verified by DNA sequencing. Ubiquitin-HA (Ub-HA) and its KR mutant were also synthesized by the same company. Duck GST-RNF34 was generated in the prokaryotic expression vector pGEX-4T-1.

shRNA negative control (shNC) and RNF34-targeting shRNAs (shRNF34), as well as siRNA negative control (siNC) and LORF2-targeting siRNAs (siLORF2), were purchased from Tsingke Biotechnology. The target sequences for shRNF34 are as follows: shRNF34–1, GCAGGTGCTAACATAGTTTGC; shRNF34–2, GGTGTTCTACTTGTCACTTGC; shRNF34–3, GGATCTTGTGCTGTGCCATCA; shRNF34–4, GCAACAGTGGTAACAGTATAA. The sequences of siLORF2 are listed below: siLORF2–1^#^: sense 5′-GGCUUGUCCAGCACUGUUA-3′, antisense 5′-UAACAGUGCUGGACAAGCC-3′; siLORF2–2^#^: sense 5′-GUAUCUGUCUGUCCUGUAA-3′, antisense 5′-UUACAGGACAGACAGAUAC-3′; siLORF2–3^#^: sense 5′-UGUCGUUAAUGUAAUGUUA-3′, antisense 5′-UAACAUUACAUUAACGACA-3′

### Virus, viral infection and TCID_50_ assay

The duck plague virus CHv strain (DPV-CHv; GenBank accession no. JQ647509) was maintained in our laboratory. Recombinant DPV expressing enhanced green fluorescent protein (DPV-CHv-GFP) was constructed using an established duck plague virus bacterial artificial chromosome (BAC) rescue system. For viral infection, DEF cells were infected with the virus at the indicated multiplicity of infection (MOI) for 2 h, washed twice with PBS, and subsequently cultured in DMEM supplemented with 2% FBS. Ultraviolet inactivated virus (DPV-UVC) was obtained by inactivating DPV-CHv-GFP under ultraviolet light for 1 hour (wavelength: 240 ~ 280 nm). Viral titers were determined by Tissue Culture Infective Dose 50 (TCID₅₀) assay. Briefly, DEF cells grown in 96-well plates were infected with 10-fold serial dilutions of virus-containing cell culture supernatants, with eight replicates per dilution. After incubation for 5–7 days at 37 °C under 5% CO₂, viral titers were calculated using the Reed-Muench method and are expressed as TCID₅₀ per milliliter.

### Real-time quantitative PCR (qPCR)

Total RNA was extracted from DEF cells or CEF cells with TRIzol Reagent (Invitrogen) following the manufacturer’s protocol. The purified RNA was then reverse-transcribed into cDNA using NovoScript Plus All-in-one 1st Strand cDNA Synthesis SuperMix (gDNA Purge; Novoprotein). Quantitative PCR was carried out with SYBR Green Master Mix (Lablead, R0202) on a Bio-Rad CFX system. Gene expression levels were normalized to RNA18S and analyzed via the 2^^(-ΔΔCT)^ method to calculate fold changes relative to the control group. DPV genomic copy numbers were quantified by absolute qPCR with primers targeting the DPV UL30 gene, as previously described [11]. All primer sequences are provided in Table 1.

**Table 1:** Primers utilized for Q-PCR.

Primers	Sequences (5’-3’)
DuIFN-β-F	TCTACAGAGCCTTGCCTGCAT
DuIFN-β-R	TGTCGGTGTCCAAAAGGATGT
DuOASL-F	TCTTCCTCAGCTGCTTCTCC
DuOASL-R	ACTTCGATGGACTCGCTGTT
DuMx-F	TGCTGTCCTTCATGACTTCG
DuMx-R	GCTTTGCTGAGCCGATTAAC
DuRNF34-F	TGACTGTTCGGCAGTTGAAG
DuRNF34-R	TTTGTGCATGTGACCATGTG
ChIFN-β-F	CCTCAACCAGATCCAGCATT
ChIFN-β-R	GGATGAGGCTGTGAGAGGAG
ChMX-F	GTTTCGGACATGGGGAGTAA
ChMX-R	GCATACGATTTCTTCAACTTTGG
ChOASL-F	CACGGCCTCTTCTACGACA
ChOASL-R	TGGGCCATACGGTGTAGACT
ChPKR-F	TGCTTGACTGGAAAGGCTACT
ChPKR-R	TCAGTCAAGAATAAACCATGTGTG
18s RNA-F	GTACAGTGAAACTGCGAATGG
18s RNA-R	CGTCGGCATGTATTAGCTCTA
DPV UL30-F	TTTCCTCCTCCTCGCTGAGTG
DPV UL30-R	CCAGAAACATACTGTGAGAGT

### Dual luciferase assay

DEF or CEF cells were seeded in 24-well plates. The transfection mixture included the firefly luciferase reporter plasmid (duck or chicken pIFNβ-Luc or pISRE-Luc), the internal control plasmid pRL-TK expressing Renilla luciferase., and expression plasmids for duck or chicken cGAS, STING, TBK1, IRF7, RNF34, and DPV LORF2. At 24 h post-transfection, the cells were stimulated with poly(dA:dT) for 12 h. Whole-cell lysates were then prepared, and luciferase activity was measured using the Dual-Glo Luciferase Assay System (Promega, E1910). Firefly luciferase activity was normalized to Renilla luciferase activity for each sample.

### Co-immunoprecipitation and western blotting

For co-immunoprecipitation (Co-IP), HEK-293T or DEF cells were transfected with the indicated plasmids for 36 h. Cells were then lysed on ice for 30 min using Western and IP lysis buffer (Beyotime, P0013) containing a protease inhibitor cocktail (TargetMol, T77081). The lysates were centrifuged at 12,000 × g for 15 min at 4 °C to remove cellular debris. A portion of the supernatant was saved as the whole-cell lysate (WCL) input control. The remaining supernatant was subjected to immunoprecipitation by incubation with target-specific antibodies overnight at 4 °C, followed by a 4-hour incubation with Protein A/G Magnetic Beads. The beads were subsequently washed five times with lysis buffer containing 1% Triton X-100. For Western blot analysis, the immunoprecipitated complexes and WCL inputs were separated by SDS-PAGE and transferred onto a PVDF membrane (Bio-Rad, 1620177). The membrane was blocked with 5% non-fat milk for 3 h at room temperature and then incubated with the corresponding primary antibody overnight at 4 °C. After washing, the membrane was incubated with an HRP-conjugated secondary antibody for 1 h at room temperature. Protein bands were visualized using Enhanced ECL Substrate (Yeasen, 36222ES60) and imaged with a ChemiDoc MP Imaging System (Bio-Rad).

### Indirect immunofluorescence assay (IFA)

DEF cells transfected with the indicated plasmids were fixed 36 h post-transfection with 4% neutral paraformaldehyde at room temperature for 20 min and then permeabilized with 0.2% Triton X-100 for 10 min. After blocking with 10% goat serum in PBS for 30 min at 37 °C, the cells were incubated with corresponding primary antibodies overnight at 4 °C. Subsequently, the cells were stained with fluorophore-conjugated secondary antibodies for 1 h at room temperature. Images were captured using an LSM 510 confocal microscope (Carl Zeiss, Jena, Germany).

### Statistical analysis

Data are presented as mean ± SEM. Differences between groups were assessed using Student’s t‑test or one‑way ANOVA followed by Tukey’s post‑hoc test. Statistical significance is indicated as follows: *, *P* < 0.05; **, *P* < 0.01; ***, *P* < 0.001; ****, *P* < 0.0001. Graph preparation and statistical analyses were performed with GraphPad Prism software (version 8.4.2; GraphPad Software, La Jolla, CA, USA).

## Results

### DuRNF34 negatively regulates the duck cGAS-STING signaling pathway

**Fig 1:**
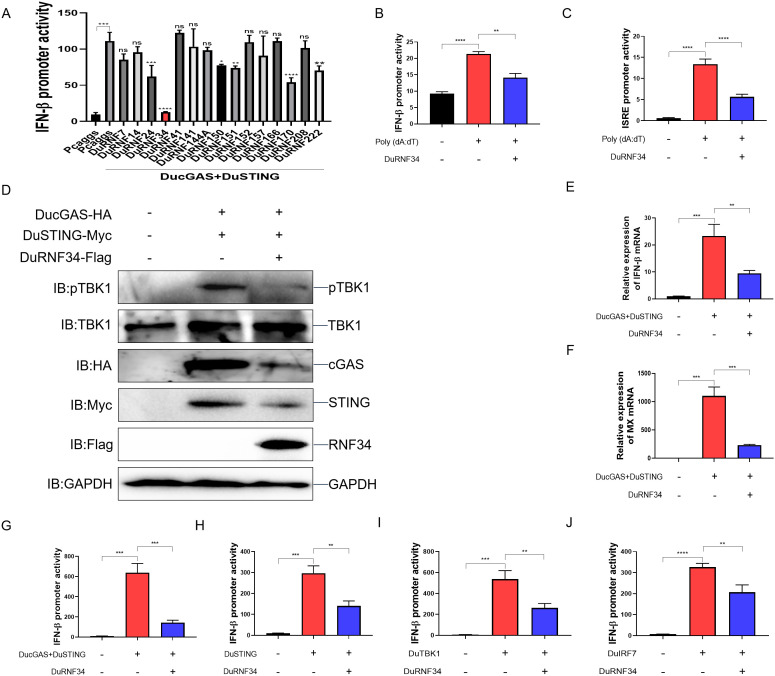
DuRNF34 negatively regulates the duck cGAS-STING signaling pathway (A) DEF cells were co-transfected with duck pIFNβ-Luc, pRL-TK, and expression plasmids for duck cGAS + STING, and indicated duck E3 ubiquitin ligases for 36 h, then harvested for luciferase assay. **(B, C)** DEF cells were co-transfected with duck pIFNβ-Luc (B) or duck pISRE-Luc (C) together with pRL-TK and DuRNF34 expression plasmid for 24 h, stimulated with poly(dA:dT) for 12 h, and then harvested for luciferase assay. **(D)** DEF cells were co-transfected with DucGAS-HA and DuSTING-Myc, with or without DuRNF34-Flag, for 36 h. WCLs were analyzed by immunoblotting. **(E, F)** DEF cells were transfected with DucGAS + DuSTING, with or without DuRNF34 expression plasmids, for 36 h. duck IFNβ (E) and duck MX (F) mRNA levels were quantified by qPCR. **(G-J)** DEF cells were co-transfected with duck pIFNβ-Luc, pRL-TK, and DuRNF34, together with DucGAS + DuSTING **(G)**, DuSTING **(H)**, DuTBK1 **(I)**, or DuIRF7 **(J)**, for 36 h, and then harvested for luciferase assay. Data are presented as mean ± standard error of the mean (SEM) and are representative of three independent experiments. Statistical significance was evaluated using two-tailed unpaired Student’s t-test. Ns, no significance; *, *P* < 0.05; **, *P* < 0.01; ***, *P* < 0.001; ****, *P* < 0.0001.

The role of avian E3 ubiquitin ligases in DNA virus infection against cGAS-STING signaling pathway remains poorly characterized. In this study, we investigated the potential involvement of duck RING-type E3 ligases in this process. Based on gene sequences predicted from the NCBI database, we attempted to amplify all duck RNF genes for the construction of eukaryotic expression vectors. However, only a subset of full-length coding sequences were successfully obtained after repeated efforts. Using these constructs, we then screened the corresponding duck RING-type E3 ligases for their ability to modulate duck IFN-β promoter activity induced by DucGAS and DuSTING. As shown in Fig 1A, duck embryo fibroblast (DEF) cells were co-transfected with a duck IFN-β promoter luciferase reporter plasmid, DucGAS and DuSTING expression plasmids, along with individual duck RING-type E3 ligase plasmids (DuRNFs). The results show that DucGAS + DuSTING strongly activated the duck IFN-β promoter, while several duck RING-type members, including DuRNF24, DuRNF34, DuRNF150, DuRNF151, DuRNF179, and DuRNF222, significantly suppressed this activation. Among these, DuRNF34 exhibited the most potent inhibitory effect, suggesting a critical role in inhibiting innate immune responses. To further define the role of DuRNF34 in DNA-triggered innate immunity, we overexpressed DuRNF34 in DEF cells and stimulated them with the dsDNA mimic poly(dA:dT). The results indicated that Poly(dA:dT) activated both the duck IFN-β and ISRE promoters, and this activation was significantly inhibited by DuRNF34 overexpression (Fig 1B and 1C). To verify the effect of DuRNF34 on duck cGAS-STING signaling, we examined DuTBK1 phosphorylation, a hallmark of innate immune activation. We found that DuRNF34 overexpression markedly suppressed DuTBK1 phosphorylation induced by DucGAS + DuSTING (Fig 1D). Consistent with this, qPCR analysis revealed that DuRNF34 significantly reduced the mRNA expression of duck IFN-β and its downstream effector duck MX induced by DucGAS + DuSTING (Fig 1E and 1F). To identify the potential target(s) of DuRNF34 within the duck cGAS-STING pathway, we co-expressed DuRNF34 with key signaling molecules (DucGAS, DuSTING, DuTBK1, and DuIRF7) in DEF cells. Dual-luciferase reporter assays demonstrated that DuRNF34 significantly suppressed duck IFN-β promoter activation induced by each of these molecules (Fig 1G-1J). Collectively, these data indicate that DuRNF34 acts as a negative regulator of the duck cGAS-STING pathway, potentially targeting DuIRF7 (the most downstream component) or multiple components.

### DuRNF34 interacts with DucGAS, DuSTING, DuTBK1, and DuIRF7

**Fig 2:**
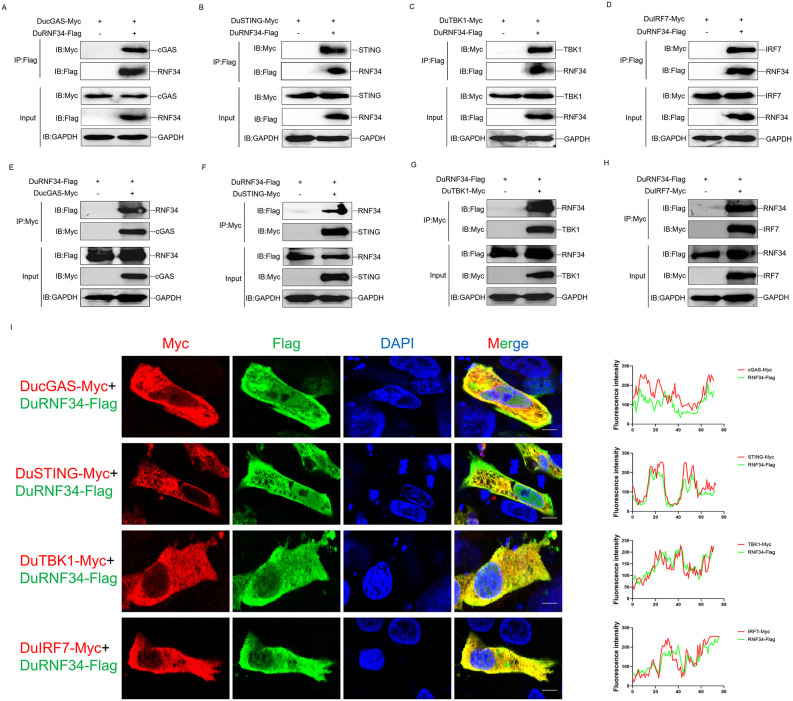
DuRNF34 interacts with DucGAS, DuSTING, DuTBK1, and DuIRF7 (A) (A-D) 293T cells were co-transfected with DucGAS-Myc (A), DuSTING-Myc (B), DuTBK1-Myc (C), DuIRF7-Myc (D) and DuRNF34-Flag plasmids for 36 h. WCLs were subjected to Co-IP with anti-Flag antibody and immunoblotting with indicated antibodies. **(E-H)** 293T cells were co-transfected with DuRNF34-Flag and DucGAS-Myc **(E)**, DuSTING-Myc **(F)**, DuTBK1-Myc **(G)**, or DuIRF7-Myc **(H)** plasmids for 36 h. WCLs were subjected to Co-IP with anti-Myc antibody and immunoblotting with indicated antibodies. **(I)** DEF cells were transfected with DuRNF34-Flag and DucGAS-Myc, DuSTING-Myc, DuTBK1-Myc, or DuIRF7-Myc plasmids for 36 h, stained with Myc (red) and Flag (green) antibodies, nuclei were stained with DAPI. The co-localization was detected by laser confocal microscopy. Scale bar, 10 μm. Fluorescence intensity in these confocal fields of vision was analyzed by Image J.

To elucidate the molecular mechanism by which DuRNF34 inhibits the duck cGAS-STING pathway, we first examined the interaction between DuRNF34 and key molecules of the pathway using co-immunoprecipitation (Co-IP) assays. As shown in Fig 2A-2D, we exogenous expressed Flag-tagged DuRNF34 and Myc-tagged DucGAS, DuSTING, DuTBK1, or DuIRF7 in cells and performed immunoprecipitation with an anti-Flag antibody. The results showed that all four immune molecules were co-precipitated with DuRNF34-Flag. Reciprocally, immunoprecipitation with an anti-Myc antibody confirmed that DuRNF34 could be pulled down with each of these molecules (Fig 2E-2H). Furthermore, confocal microscopy revealed clear cytoplasmic co-localization of DuRNF34 with DucGAS, DuSTING, DuTBK1, and DuIRF7 in DEF cells (Fig 2I). Together, these results demonstrate that DuRNF34 physically interacts with multiple core components of the duck cGAS-STING signaling pathway.

### DuRNF34 promotes the degradation of DucGAS, DuSTING, and DuIRF7 via multiple cellular pathways

**Fig 3:**
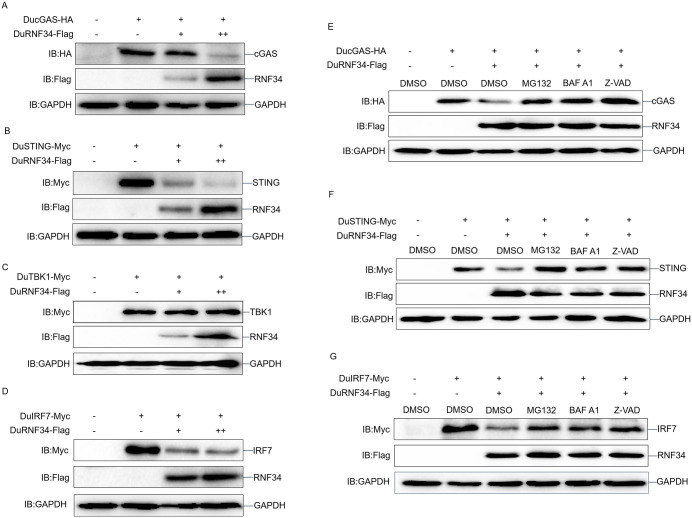
DuRNF34 promotes the degradation of DucGAS, DuSTING, and DuIRF7 via multiple cellular pathways. **(A-D)** DEF cells were co-transfected with DuRNF34-Flag and DucGAS-HA **(A)**, DuSTING-Myc **(B)**, DuTBK1-Myc **(C)**, or DuIRF7-Myc (D) for 36 h, and WCLs were analyzed by immunoblotting. **(E-G)** DEF cells were co-transfected with DuRNF34-Flag and DucGAS-HA **(E)**, DuSTING-Myc **(F)**, or DuIRF7-Myc **(G)** for 24 h, treated with inhibitors for 12 h, and then analyzed by immunoblotting.

To further explore how avian RNF34 regulates these molecules, we co-expressed DuRNF34 with DucGAS, DuSTING, DuTBK1, or DuIRF7 in DEF cells. We found that DuRNF34 overexpression resulted in a significant decrease in the protein levels of DucGAS, DuSTING and DuIRF7 in a dose-dependent manner, whereas DuTBK1 expression remained largely unaffected (Fig 3A-3D). To determine which degradation pathways are involved in DuRNF34-mediated protein reduction, we treated DEF cells co-expressing DuRNF34 with DucGAS, DuSTING, or DuIRF7 using the proteasome inhibitor MG132, the autophagy inhibitor BAF A1, or the apoptosis inhibitor Z-VAD. The results showed that each of these inhibitors effectively rescued the decreased protein levels of DucGAS, DuSTING, and DuIRF7 induced by DuRNF34 (Fig 3E-3G). Together, these findings indicate that DuRNF34 mediates the degradation of DucGAS, DuSTING, and DuIRF7 through multiple pathways, thereby inhibiting the duck cGAS-STING pathway-mediated innate immune response.

To further investigate whether the immune suppressive function of avian RNF34 is evolutionarily conserved in avian species, we cloned and overexpressed chicken RNF34 (ChRNF34) in chicken embryo fibroblast (CEF) cells stimulated with Poly(dA:dT). The results showed that ChRNF34 inhibited Poly(dA:dT) induced activation of both the chicken IFN-β and chicken ISRE promoters in a dose-dependent manner (S1A and S1B Fig), suggesting an immune inhibitory function analogous to that of its duck ortholog. We next examined whether ChRNF34 influences key components of the chicken cGAS-STING pathway by co-expressing ChRNF34 with ChcGAS, ChSTING, ChTBK1, or ChIRF7 in CEF cells. We found that only ChRNF34 promoted the degradation of ChIRF7 in a dose-dependent manner but did not alter the protein levels of ChcGAS, ChSTING, or ChTBK1 (S1C-S1F Fig), indicating that ChRNF34 specifically targets ChIRF7. To determine which degradation pathways are involved in ChRNF34-mediated protein reduction, we treated CEF cells co-expressing ChRNF34 with ChIRF7 using the proteasome inhibitor MG132, the autophagy inhibitor BAF A1, or the apoptosis inhibitor Z-VAD. The results showed that, similar to the duck system, each of these inhibitors effectively rescued the decreased protein levels of ChIRF7 induced by ChRNF34 (S1G Fig). To investigate the interaction between ChRNF34 and ChIRF7, we co-expressed ChRNF34-Myc and ChIRF7-Flag in CEF cells. Co-IP assays were then performed using antibodies against both the Flag and Myc tags. The results indicated that ChRNF34 and ChIRF7 could be co-precipitated with each other (S1H and S1I Fig), demonstrating a direct physical interaction between these two proteins. To confirm that ChRNF34 exerts its immunosuppressive effect via ChIRF7, we co-transfected ChRNF34 and ChIRF7 into CEF cells. Dual-luciferase reporter assays demonstrated that ChRNF34 significantly inhibited ChIRF7 induced activation of the chicken IFN-β and chicken ISRE promoters (S1J and S1K Fig). Consistent with this, qPCR results showed that ChRNF34 markedly reduced the mRNA expression of chicken IFN-β, MX, OASL, and PKR induced by ChIRF7 (S1L-S1O Fig). In summary, these results demonstrate that the immune inhibitory function of RNF34 on the cGAS-STING pathway is highly conserved in avian evolution.

### DuRNF34 catalyzes the ubiquitination and degradation of DucGAS, DuSTING, and DuIRF7

**Fig 4:**
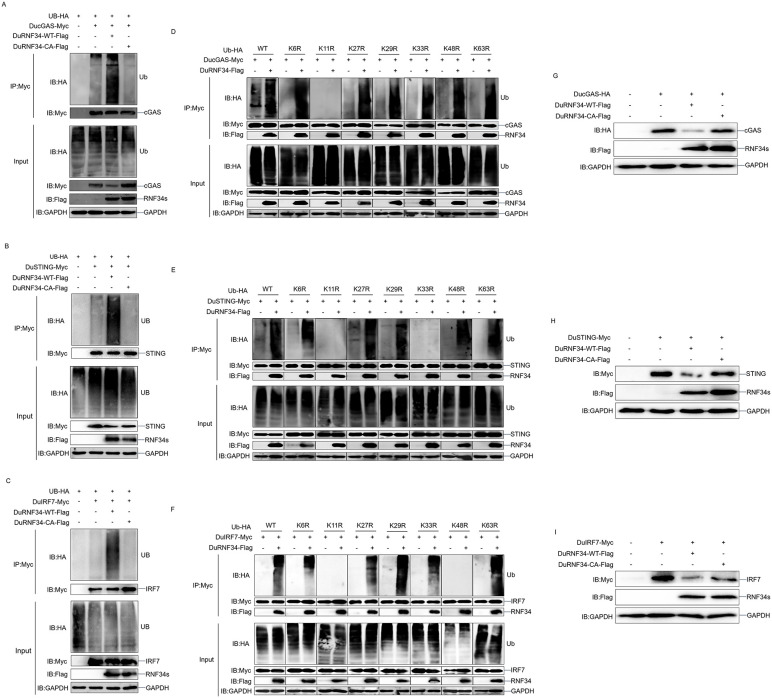
DuRNF34 catalyzes the ubiquitination and degradation of DucGAS, DuSTING, and DuIRF7 (A-C) DEF cells were co-transfected with Ub-HA, DuRNF34-WT/CA-Flag, and DucGAS-Myc (A), DuSTING-Myc (B), or DuIRF7-Myc (C) expression plasmids for 24 h, then treated with inhibitors for 12 h, and WCLs were IPed with anti-Myc antibody and analyzed by immunoblotting. **(D-F)** DEF cells were transfected with Ub-WT/KR-HA and DuRNF34-Flag, together with DucGAS-Myc **(D)**, DuSTING-Myc **(E)**, or DuIRF7-Myc **(F)** plasmids for 24 h, then treated with inhibitors for 12 h, and WCLs were IPed with anti-Myc antibody and analyzed by immunoblotting. **(G-I)** DEF cells were co-transfected with DuRNF34-WT/CA-Flag and DucGAS-HA **(G)**, DuSTING-Myc **(H)**, or DuIRF7-Myc **(I)** expression plasmids for 36 h, and WCLs were analyzed by immunoblotting.

Post-translational modifications (PTMs) play a critical role in PRRs-dependent innate immune responses by targeting innate sensors and downstream signaling molecules [46,47]. Among these PTMs, ubiquitination is one of the most extensively studied. Ubiquitin is a 76-amino-acid protein containing seven lysine residues (K6, K11, K27, K29, K33, K48, K63), which serve as linkage sites for the assembly of polyubiquitin chains [48]. Since DuRNF34 is an E3 ubiquitin ligase, we next examined whether it promotes the ubiquitination of DucGAS, DuSTING, and DuIRF7. We generated a catalytically inactive DuRNF34 mutant (DuRNF34-CA) by mutating Cys 309 and His 323 to Ala. In DEF cells, we co-transfected with Myc-tagged DucGAS, DuSTING, or DuIRF7, together with Flag-tagged wild-type DuRNF34 (DuRNF34-WT) or DuRNF34-CA and HA-tagged ubiquitin, immunoprecipitation assays showed that DuRNF34-WT, but not DuRNF34-CA, significantly enhanced the ubiquitination of all three proteins (Fig 4A-4C). These results indicate that DuRNF34 facilitates the ubiquitination of DucGAS, DuSTING, and DuIRF7 in a ligase activity-dependent manner. We then sought to identify the types of polyubiquitin chains catalyzed by DuRNF34. A series of ubiquitin mutants were constructed in which each of the seven Lys residues (K6, K11, K27, K29, K33, K48, K63) was individually mutated to Arg (K-to-R). Ubiquitination assays revealed that the Ub-K11R mutation completely abolished DuRNF34-mediated ubiquitination of DucGAS (Fig 4D). For DuSTING, ubiquitination was abolished by the Ub-K11R and Ub-K33R mutations (Fig 4E), while for DuIRF7, Ub-K11R and Ub-K48R mutations eliminated ubiquitination (Fig 4F). These findings demonstrate that DuRNF34 promotes K11-linked polyubiquitination of DucGAS, K11-/K33-linked polyubiquitination of DuSTING, and K11-/K48-linked polyubiquitination of DuIRF7. To further confirm the role of DuRNF34’s ligase activity in protein degradation, we co-transfected DEF cells with DuRNF34-WT or DuRNF34-CA together with DucGAS, DuSTING, or DuIRF7. The results showed that compared to DuRNF34-WT, the catalytically inactive DuRNF34-CA mutant exhibited a significantly impaired ability to promote the degradation of all three signaling molecules (Fig 4G-4I). This confirms that DuRNF34-mediated degradation of DucGAS, DuSTING, and DuIRF7 is directly dependent on its ubiquitin ligase function.

### DPV exploits DuRNF34 to antagonize antiviral immunity by promoting DuIRF7 degradation

**Fig 5:**
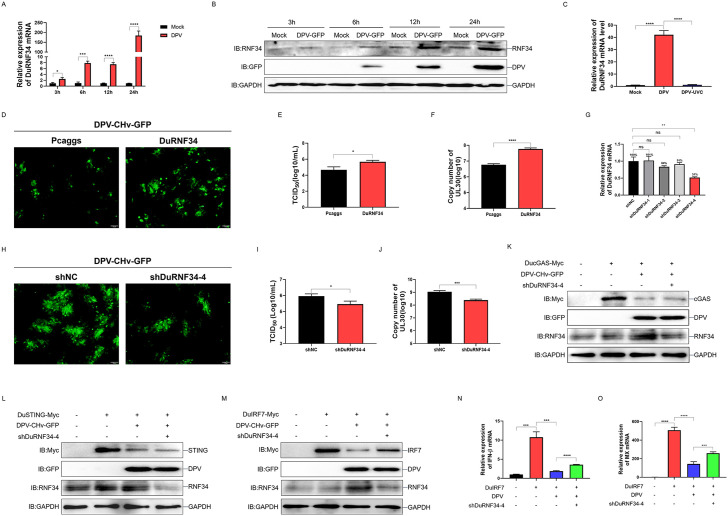
DPV exploits DuRNF34 to antagonize antiviral immunity by promoting DuIRF7 degradation. **(A, B)** DEF cells were infected with 0.1MOI of DPV-CHv-GFP and harvested at indicated time points to quantify DuRNF34 mRNA levels by qPCR and DuRNF34 protein levels by immunoblotting. **(C)** DEF cells were infected with live virus DPV-CHv-GFP or UV-inactivated DPV-CHv-GFP (DPV-UVC) at 0.1 MOI, respectively. Cells were collected 24 hours after infection, and DuRNF34 mRNA levels were quantified by qPCR. **(D-F)** DEF cells were transfected with Pcaggs or DuRNF34 expression plasmids for 12h, and then infected with DPV-CHv-GFP at 0.1MOI for 24h. Virus fluorescent spots were collected by fluorescence microscope, the scale bar is 100 μm **(D)**, the supernatant was collected to detect virus titer **(E)**, and the cells were collected to detect virus copy number **(F)**. **(G)** DEF cells were transfected with shRNA for 36 h, and DuRNF34 mRNA levels were quantified by qPCR. **(H-J)** DEF cells were transfected with shNC or shDuRNF34-4 for 12h, and then infected with DPV-CHv-GFP at 0.1MOI for 24h. Virus fluorescent spots were collected by fluorescence microscope, the scale bar is 100 μm **(H)**, the supernatant was collected to detect virus titer **(I)**, and the cells were collected to detect virus copy number **(J)**. **(K-M)** DEF cells were transfected with shRNA and DucGAS-Myc **(K)**, DuSTING-Myc **(L)** or DuIRF7-Myc **(M)** for 12h, and then infected with DPV-CHv-GFP at 0.1MOI for 24h, and WCLs were analyzed by immunoblotting. **(N, O)** DEF cells were transfected with shRNA and DuIRF7-Myc for 12h, and then infected with DPV-CHv-GFP at 0.1MOI for 24h. duck IFNβ **(N)** and duck MX **(O)** mRNA levels were quantified by qPCR. Data are presented as mean ± standard error of the mean (SEM) and are representative of three independent experiments. Statistical significance was evaluated using two-tailed unpaired Student’s t-test. Ns, no significance; *, *P* < 0.05; **, *P* < 0.01; ***, *P* < 0.001; ****, *P* < 0.0001.

To investigate the role of DuRNF34 during DPV infection, we first examined whether DPV infection influences DuRNF34 expression in DEF cells. qPCR and immunoblotting analyses revealed that DPV significantly upregulated DuRNF34 expression at both the mRNA (Fig 5A) and protein (Fig 5B) levels, suggesting that DuRNF34 is involved in the host cellular response to DPV infection. To determine whether the upregulation of DuRNF34 is triggered by viral particle entry or requires active viral replication, we infected DEF cells with either live DPV or ultraviolet inactivated DPV (DPV-UVC) and measured DuRNF34 mRNA levels 24 h post-infection. The results showed that live DPV infection significantly increased DuRNF34 mRNA, whereas DPV-UVC infection did not induce a substantial upregulation (Fig 5C). These data indicate that the induction of DuRNF34 expression during DPV infection depends on active viral replication. Based on this finding, we further evaluated the functional consequence of DuRNF34 on viral replication. DEF cells overexpressing DuRNF34 were infected with DPV-CHv-GFP at a multiplicity of infection (MOI) of 0.1. Subsequent analysis showed that compared with the control group, DuRNF34 overexpression significantly enhanced the intensity of viral fluorescent spots (Fig 5D), increased viral titers (Fig 5E), and elevated the copy number of the viral genome (Fig 5F). To further delineate the role of DuRNF34, we performed DuRNF34 knockdown in DEF cells by transfecting shRNA targeting DuRNF34. Among the tested shRNAs, shDuRNF34–4 showed a significant knockdown effect (Fig 5G). In contrast to the overexpression results, DPV infection of DuRNF34-knockdown DEF cells revealed that DuRNF34 knockdown markedly reduced DPV fluorescent plaque intensity, viral titer, and viral genome copy number (Fig 5H-5J), confirming that DuRNF34 promotes DPV proliferation in DEF cells. To determine whether this effect of DuRNF34 is associated with its immune suppressive function, we overexpressed DucGAS, DuSTING, or DuIRF7 in DuRNF34-knockdown DEF cells followed by DPV infection. We found that DPV infection induced significant degradation of DucGAS, DuSTING, and DuIRF7 protein levels, while DuRNF34 knockdown specifically attenuated DPV mediated degradation of DuIRF7, but not that of DucGAS or DuSTING (Fig 5K-5M). Correspondingly, qPCR results demonstrated that DPV infection substantially suppressed the mRNA levels of DuIRF7-induced duck IFNβ and duck MX, and this suppression was alleviated by DuRNF34 knockdown (Fig 5N and 5O). These results suggest that DPV exploits DuRNF34 to selectively target and degrade DuIRF7, thereby inhibiting the antiviral immune response and facilitating viral proliferation.

### DPV LORF2 suppresses innate immunity by targeting DuIRF7 for degradation

**Fig 6:**
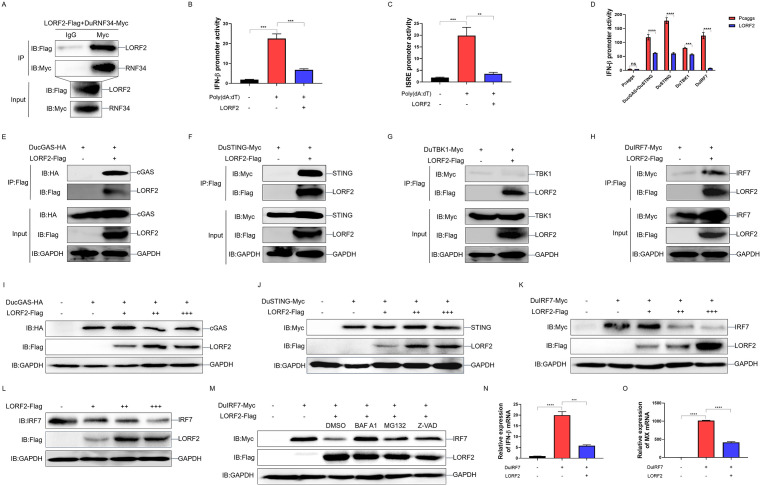
DPV LORF2 suppresses innate immunity by targeting DuIRF7 for degradation. **(A)** 293T cells were co-transfected with LORF2-Flag and DuRNF34-Myc expression plasmids for 36 **h.** WCLs were subjected to Co-IP with anti-Myc antibody and immunoblotted with the indicated antibodies. **(B, C)** DEF cells were co-transfected with duck pIFNβ-Luc (B) or duck pISRE-Luc (C) together with pRL-TK and LORF2 expression plasmids for 24 h, stimulated with poly(dA:dT) for 12 h, and then harvested for luciferase assay. **(D)** DEF cells were co-transfected with duck pIFNβ-Luc, pRL-TK, and LORF2, together with DucGAS + DuSTING, DuSTING, DuTBK1, or DuIRF7 expression plasmids for 36 h, and then harvested for luciferase assay. **(E-H)** 293T cells were co-transfected with LORF2-Flag and DucGAS-HA **(E)**, DuSTING-Myc **(F)**, DuTBK1-Myc **(G)**, or DuIRF7-Myc (H) for 36 **h.** WCLs were used for Co-IP with anti-Flag antibody and immunoblotting with the indicated antibodies. **(I-K)** DEF cells were co-transfected with DuRNF34-Flag and DucGAS-HA **(I)**, DuSTING-Myc **(J)**, or DuIRF7-Myc (K) for 36 h. WCLs were analyzed by immunoblotting. **(L)** DEF cells were transfected with increasing amounts of DuRNF34-Flag expression plasmid for 36 h. WCLs were analyzed by immunoblotting. **(M)** DEF cells were co-transfected with DuRNF34-Flag and DuIRF7-Myc expression plasmids for 24 h, treated with inhibitors for 12 h, and then analyzed by immunoblotting. **(N, O)** DEF cells were transfected with DuIRF7 together with or without LORF2 expression plasmid for 36 h. duck IFNβ **(N)** and duck MX **(O)** mRNA levels were quantified by qPCR. Data are presented as mean ± standard error of the mean (SEM) and are representative of three independent experiments. Statistical significance was evaluated using two-tailed unpaired Student’s t-test. Ns, no significance; *, *P* < 0.05; **, *P* < 0.01; ***, *P* < 0.001; ****, *P* < 0.0001.

To establish a direct link between DuRNF34 and DPV viral proteins, DuRNF34 protein fused to GST (DuRNF34-GST) was expressed using the prokaryotic vector pGEX-4T-1 in *E. coli* BL21 and then purified it using glutathione agarose (S2A Fig). The purified DuRNF34-GST protein was incubated with lysates from DPV-infected DEF cells, and potential interacting viral partners were identified through affinity purification coupled with mass spectrometry (AP-MS). The MS results revealed that several DPV proteins, including US2, LORF2, LORF4, UL2, UL17, UL25 and UL31, as potential binding partners of DuRNF34 (S2B Fig). To validate these interactions, we co-transfected cells with DuRNF34 and eukaryotic expression plasmids encoding each of the candidate DPV proteins, followed by Co-IP assays. The results showed that DuRNF34 co-precipitated with US2 and LORF2 (S2C and S2D Fig), but not with the other viral proteins tested (S2E-S2I Fig), indicating that DuRNF34 interacts with US2 and LORF2. Among these interacting partners, LORF2, a DPV unique protein whose function was previously uncharacterized, attracted particular attention. To further confirm the interaction between DuRNF34 and LORF2, cells were co-transfected with LORF2-Flag and DuRNF34-Myc expression plasmids, and immunoprecipitation was performed using an anti-Myc antibody. The results demonstrated that LORF2 was also co-precipitated with DuRNF34 (Fig 6A), confirming a specific physical interaction between the two proteins. To evaluate the role of LORF2 in innate immunity, we ectopically expressed LORF2 in DEF cells stimulated with poly(dA:dT). The results showed that poly(dA:dT) treatment significantly activated both the duck IFN-β and duck ISRE promoters, whereas LORF2 expression markedly suppressed this activation (Fig 6B and 6C), indicating that LORF2 broadly suppresses DNA-triggered innate immune signaling. We further clarified the role of LORF2 in the duck cGAS-STING pathway by co-expressing LORF2 with key signaling components (DucGAS, DuSTING, DuTBK1, or DuIRF7) in DEF cells. Luciferase reporter assays demonstrated that LORF2 markedly inhibited duck IFN-β promoter activity induced by these immune factors (Fig 6D), indicating that LORF2 suppresses duck cGAS-STING-mediated IFN-β production. Co-IP assays were performed to identify the direct target(s) of LORF2. The results revealed that LORF2 interacts with DucGAS, DuSTING, and DuIRF7, but not with DuTBK1 (Fig 6E-6H). To determine whether LORF2 affects the stability of these signaling molecules, we examined protein levels upon LORF2 expression. The results showed that LORF2 promoted the degradation of DuIRF7 but did not affect the expression of DucGAS or DuSTING (Fig 6I-6K). This targeting of DuIRF7 by LORF2 was further confirmed by observing a decrease in endogenous DuIRF7 levels (Fig 6L). We next investigated the degradation pathway involved. DEF cells expressing LORF2 and DuIRF7 were treated with the proteasome inhibitor MG132, the autophagy inhibitor BAF A1, or the apoptosis inhibitor Z-VAD. We observed that all three inhibitors reversed DuIRF7 degradation induced by LORF2 (Fig 6M), which aligns with the observed role of DuRNF34. Furthermore, qPCR analysis showed that LORF2 significantly reduced the mRNA levels of duck IFN-β and duck MX induced by DuIRF7 (Fig 6N and 6O). Together, these findings demonstrate that the DPV protein LORF2 specifically interacts with DuRNF34 and targets DuIRF7 for degradation through multiple cellular pathways, thereby effectively suppressing the host innate antiviral response.

To investigate whether the other interacting protein US2 affects the antiviral activity of the cGAS-STING pathway, we employed the dual luciferase reporter gene assay. We found that the overexpression of DPV US2 significantly inhibited the activation of the IFN-β promoter mediated by the cGAS-STING pathway (S2J Fig). This result indicates that US2 also inhibits the cGAS-STING immune signaling pathway.

### DPV LORF2 recruits DuRNF34 to promote DuIRF7 ubiquitination and degradation

**Fig 7:**
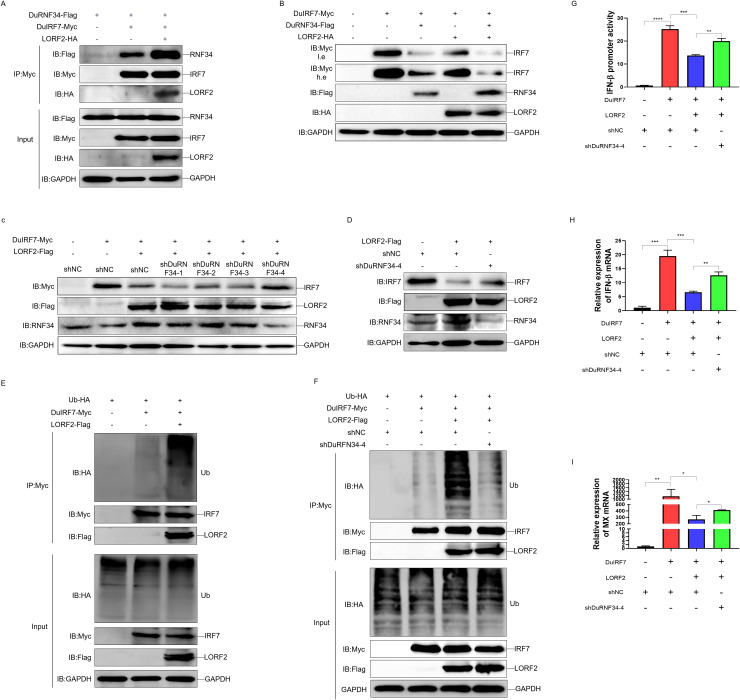
DPV LORF2 recruits DuRNF34 to promote DuIRF7 ubiquitination and degradation. **(A)** DEF cells were co-transfected with DuRNF34-Flag, DuIRF7-Myc, and LORF2-HA expression plasmids for 24 h, treated with inhibitors for 12 h, and WCLs were IPed with anti-Myc antibody and analyzed by immunoblotting. **(B)** DEF cells were co-transfected with DuIRF7-Myc, DuRNF34-Flag, and LORF2-HA expression plasmids for 36 h. WCLs were analyzed by immunoblotting. **(C)** DEF cells were transfected with DuIRF7-Myc, LORF2-Flag, and shRNA for 36 h. WCLs were analyzed by immunoblotting. **(D)** DEF cells were transfected with LORF2-Flag and shRNA for 36 h. WCLs were analyzed by immunoblotting. **(E)** DEF cells were co-transfected with Ub-HA, DuIRF7-Myc, and LORF2-Flag expression plasmids for 24 h, treated with inhibitors for 12 h, and WCLs were IPed with anti-Myc antibody and immunoblotting with the indicated antibodies. **(F)** DEF cells were transfected with the indicated plasmids for 24 h, treated with inhibitors for 12 h, and WCLs were IPed with anti Myc antibody and immunoblotting with the indicated antibodies. **(G)** DEF cells were co-transfected with duck pIFNβ-Luc, pRL-TK, and the indicated plasmids for 36 h, and then harvested for luciferase assay. **(H, I)** DEF cells were co-transfected with the indicated plasmids for 36 h, and duck IFNβ (H) and duck MX (I) mRNA levels were quantified by qPCR. Data are presented as mean ± standard error of the mean (SEM) and are representative of three independent experiments. Statistical significance was evaluated using two-tailed unpaired Student’s t-test. Ns, no significance; *, *P* < 0.05; **, *P* < 0.01; ***, *P* < 0.001; ****, *P* < 0.0001.

To determine how LORF2 exploits DuRNF34 to regulate DuIRF7, we first examined the effect of LORF2 on the interaction between DuRNF34 and DuIRF7 by Co-IP. The results showed that LORF2 strengthened the binding between DuRNF34 and DuIRF7 (Fig 7A). We next assessed the combined effect of LORF2 and DuRNF34 on DuIRF7 protein levels. We found that both LORF2 and DuRNF34 individually promoted DuIRF7 degradation, and their co-expression resulted in a more pronounced reduction in DuIRF7 abundance (Fig 7B). To investigate whether this degradation is cell type-specific, we co-expressed DuIRF7, DuRNF34 and LORF2 in Duck Embryo Hepatocytes (DEH) cells. The results were consistent with those of the DEF cells. Both DuRNF34 and LORF2 could promote the degradation of DuIRF7, and when they were present together, the degradation ability was even stronger (S3 Fig). To investigate whether DuRNF34 is required for LORF2-mediated DuIRF7 degradation, we transfected DuIRF7 and LORF2 in DEF cells knockdown of DuRNF34. The results showed that the degradation of exogenous expressed DuIRF7 induced by LORF2 was significantly impaired in shDuRNF34–4 mediated DuRNF34 knockdown cells (Fig 7C). Similarly, knockdown of DuRNF34 also attenuated LORF2-induced degradation of endogenous DuIRF7 (Fig 7D), confirming that LORF2-mediated DuIRF7 degradation depends on DuRNF34. We further investigated whether LORF2 influences DuIRF7 ubiquitination. DEF cells were co-transfected with UB-HA, DuIRF7-Myc, and LORF2-Flag, followed by immunoprecipitation using a Myc antibody. The results demonstrated that LORF2 promoted the ubiquitination of DuIRF7 (Fig 7E). To determine whether this ubiquitination was dependent on DuRNF34, we transfected UB-HA, DuIRF7-Myc, and LORF2-Flag in DuRNF34-knockdown DEF cells. The results showed that DuRNF34 knockdown significantly inhibited LORF2-induced ubiquitination of DuIRF7 (Fig 7F), indicating that LORF2-mediated ubiquitination of DuIRF7 requires DuRNF34. To further determine whether DuRNF34 is involved in LORF2-mediated immune suppression, we transfected RNF34-knockdown DEF cells with DuIRF7 and LORF2. Dual-luciferase reporter and qPCR assays revealed that LORF2 significantly inhibited DuIRF7-induced duck IFN-β promoter activity, and reduced mRNA levels of duck IFN-β and duck MX. Notably, this inhibitory effect was substantially attenuated upon DuRNF34 knockdown (Fig 7G-7I). These results demonstrate that DPV LORF2 recruits DuRNF34 to ubiquitinate and degrade DuIRF7, thereby suppressing the innate immune response.

**Fig 8:**
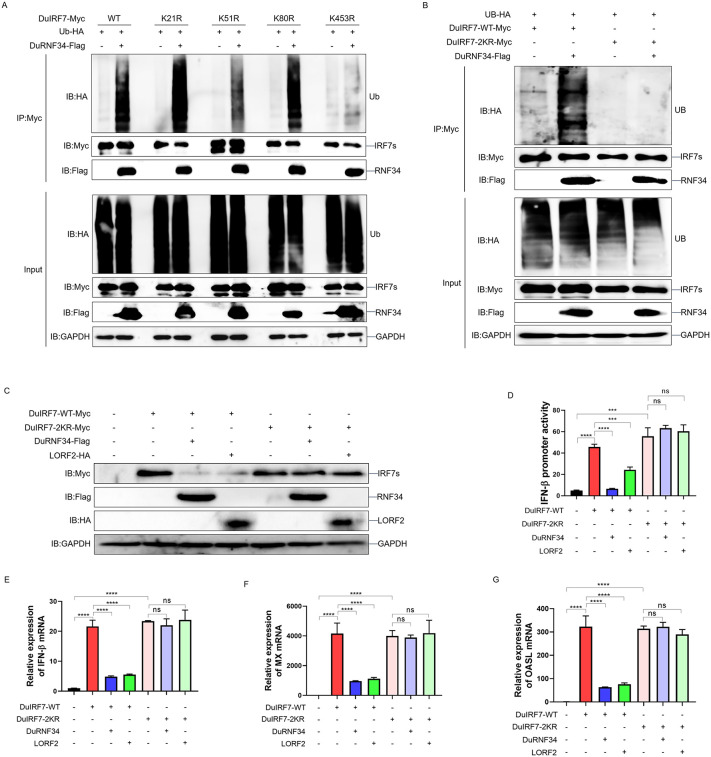
DuRNF34 catalyzes the ubiquitination of DuIRF7 at K51 and K453. **(A, B)** DEF cells were co-transfected with Ub-HA, DuIRF7-WT/KR-Myc, and DuRNF34-Flag expression plasmids for 24 h, treated with inhibitors for 12 h. WCLs were IPed with anti-Myc antibody and analyzed by immunoblotting. **(C)** DEF cells were co-transfected with DuIRF7-WT/2KR-Myc, DuRNF34-Flag, and LORF2-HA expression plasmids for 36 h. WCLs were analyzed by immunoblotting. **(D)** DEF cells were co-transfected with duck pIFNβ-Luc, pRL-TK, and the indicated plasmids for 36 h, and then harvested for luciferase assay. **(E-G)** DEF cells were co-transfected with the indicated plasmids for 36 h, and duck IFNβ **(E)**, duck MX **(F)**, and duck OASL **(G)** mRNA levels were quantified by qPCR. Data are presented as mean ± standard error of the mean (SEM) and are representative of three independent experiments. Statistical significance was evaluated using two-tailed unpaired Student’s t-test. Ns, no significance; *, *P* < 0.05; **, *P* < 0.01; ***, *P* < 0.001; ****, *P* < 0.0001.

To identify the specific ubiquitination sites on DuIRF7 targeted by the LORF2 recruited DuRNF34, we performed immunoprecipitation coupled with mass spectrometry (IP-MS) in DEF cells co-expressing DuRNF34-Flag and DuIRF7-Myc. MS analysis identified that K21, K51, K80, and K453 on DuIRF7 were potential ubiquitination sites. To verify these sites, we constructed single Lys-to-Arg (K-to-R) point mutants of DuIRF7 (K21R, K51R, K80R, K453R) and assessed their ubiquitination in the presence of DuRNF34. The results showed that ubiquitination of DuIRF7 by DuRNF34 was significantly reduced upon mutation of K51 or K453 (Fig 8A). To further clarify the role of these two sites, we constructed a double mutant of DuIRF7 (DuIRF7–2KR) in which both K51 and K453 were mutated to Arg. Ubiquitination assays showed that DuRNF34-mediated ubiquitination of DuIRF7 was completely abolished in the DuIRF7–2KR mutant (Fig 8B), indicating that K51 and K453 are the key ubiquitination sites targeted by the LORF2 recruited DuRNF34 complex. We next investigated whether these two sites are responsible for LORF2 and DuRNF34 induced DuIRF7 degradation. DEF cells were transfected with either DuIRF7-WT or the DuIRF7–2KR mutant, together with LORF2 and DuRNF34. The results demonstrated that LORF2 and RNF34 significantly promoted the degradation of DuIRF7-WT, while they had no effect on the stability of the DuIRF7–2KR mutant (Fig 8C). Consistently, Dual-luciferase reporter assays and qPCR results showed that LORF2 and DuRNF34 significantly suppressed duck IFN-β promoter activity and the mRNA expression of duck IFN-β, MX, and OASL induced by DuIRF7-WT, whereas these inhibitory effects were absent in DuIRF7–2KR expressing cells (Fig 8D-8G). Collectively, these data demonstrate that DPV LORF2 recruits DuRNF34 to catalyze DuIRF7 ubiquitination at K51 and K453, leading to DuIRF7 degradation and suppression of DuIRF7-driven innate immune responses.

### LORF2 is essential for viral proliferation and immune evasion during DPV infection

**Fig 9:**
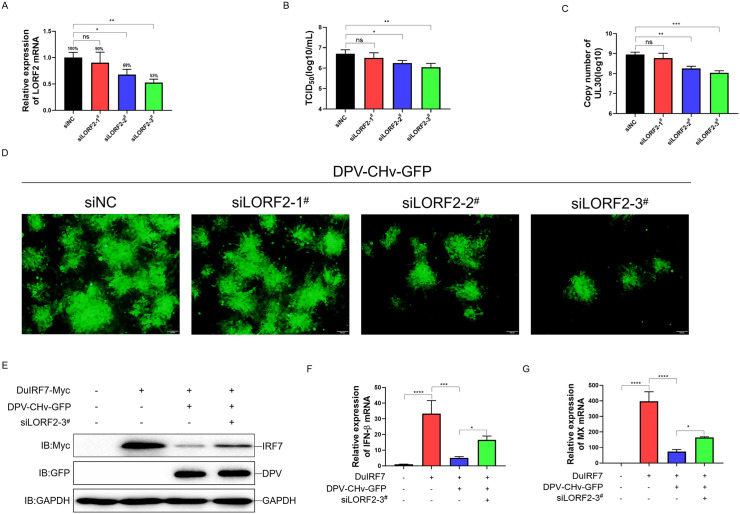
LORF2 is essential for viral proliferation and immune evasion during DPV infection. **(A-D)** DEF cells were infected with DPV-CHv-GFP at 0.1 MOI and transfected with siRNA for 24 h. The mRNA level of LORF2 was measured by qPCR **(A)**. The supernatant was collected for viral titer detection **(B)**, and cells were collected for viral copy number quantification **(C)**. Viral fluorescent spots were observed by fluorescence microscopy, scale bar is 100 μm **(D)**. **(E-G)** DEF cells were transfected with DuIRF7-Myc for 12 h, then infected with DPV-CHv-GFP at 0.1 MOI and transfected with siRNA for 24 h. WCLs were analyzed by immunoblotting **(E)**, duck IFNβ **(F)** and duck MX **(G)** mRNA levels were quantified by qPCR. Data are presented as mean ± standard error of the mean (SEM) and are representative of three independent experiments. Statistical significance was evaluated using two-tailed unpaired Student’s t-test. Ns, no significance; *, *P* < 0.05; **, *P* < 0.01; ***, *P* < 0.001; ****, *P* < 0.0001.

To investigate the functional role of LORF2 during DPV infection, we knocked down LORF2 expression in DPV-CHv-GFP-infected DEF cells using siRNA. qPCR analysis confirmed that siLORF2–3^#^ could most significantly reduce LORF2 expression (Fig 9A). We next examined the effect of LORF2 on viral replication by infecting DEF cells with DPV-CHv-GFP at an MOI of 0.1 and transfecting with siLORF2. We found that LORF2 knockdown significantly reduced viral titer, viral genome copies and viral plaque intensity (Fig 9B-9D), indicating that LORF2 supports DPV proliferation in DEF cells. To assess whether LORF2 influences DuIRF7 stability during viral infection, we transfected DEF cells with DuIRF7 and siRNA, followed by infection with DPV-CHv-GFP. The results showed that DPV infection strongly promoted DuIRF7 degradation in siNC treated cells. In contrast, this degradation was markedly attenuated in siLORF2 treated cells (Fig 9E), suggesting that LORF2 is essential for DuIRF7 degradation during DPV infection. Consistently, qPCR results showed that DPV infection significantly suppressed DuIRF7 induced mRNA expression of duck IFN-β and duck MX, and this suppression was significantly attenuated by LORF2 knockdown (Fig 9F and 9G). These results demonstrate that LORF2 promotes DPV replication in DEF cells by degrading DuIRF7, thereby inhibiting DuIRF7 mediated antiviral innate immunity.

## Discussion

**Fig 10:**
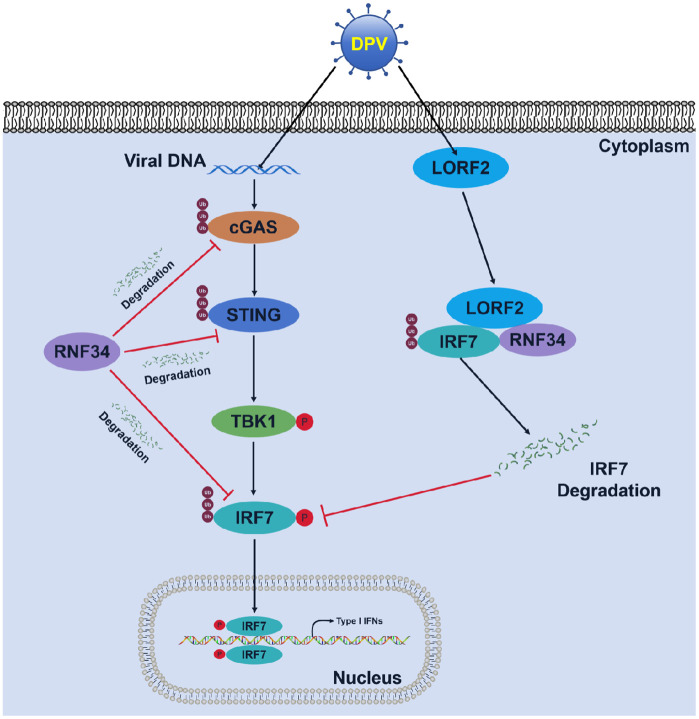
A working model of DPV LORF2 recruiting RNF34 to inhibit IRF7-mediated antiviral innate immunity. The host E3 ubiquitin ligase DuRNF34 interacts with and promotes the ubiquitination and degradation of DucGAS, DuSTING, and DuIRF7, thereby inhibiting IFN production. DPV co-opts LORF2 to recruit DuRNF34 specifically targeting DuIRF7 for ubiquitination-mediated degradation, thereby facilitating viral proliferation.

Innate immune signaling pathways play a critical role in antiviral defense. To ensure survival and replication, herpesviruses have evolved diverse strategies to evade these antiviral mechanisms. For instance, the HSV-1 capsid protein UL38 directly binds STING, preventing cGAMP binding and disrupting the STING-TBK1-IRF3 complex, thereby suppressing IFN and downstream antiviral gene expression [49]. Similarly, Pseudorabies virus (PRV) UL13 inhibits cGAS-STING-mediated IFN-β production by phosphorylating IRF3 [50]. In this study, we elucidated the immune escape mechanism by which the DPV unique protein LORF2 recruited the duck host E3 ubiquitin ligase RNF34 to promote duck IRF7 ubiquitination and degradation, thereby inhibiting duck IRF7-mediated antiviral innate immune response (Fig 10). Specifically, we identified duck RNF34 as a negative regulator of the duck cGAS-STING signaling pathway, which targets multiple core components of the pathway, including cGAS, STING, TBK1, and IRF7, and mediates the degradation of cGAS, STING, and IRF7 in an E3 ubiquitin ligase activity dependent manner. Notably, DPV infection upregulates duck RNF34 expression, and its overexpression enhances viral proliferation, whereas duck RNF34 knockdown attenuates DPV induced degradation of duck IRF7 and suppresses viral replication. Further AP-MS screening assays revealed that DPV co-opts LORF2 to recruit duck RNF34, selectively promoting the degradation of duck IRF7 and enhancing viral immune evasion. Functional experiments confirmed that knockdown of LORF2 significantly inhibited DPV induced duck IRF7 degradation and reduced viral replication in DEF cells. This study establishes the first link between avian RNF34 and DNA virus infection against the cGAS-STING pathway, uncovering a novel immune evasion strategy employed by DPV. These insights may provide a new potential target for future antiviral strategies and vaccine design.

Avian E3 ubiquitin ligases have been shown to play significant and diverse roles in the process of avian virus infection. This process involves the attachment of ubiquitin molecular chains to viral proteins, thereby marking them for degradation. For instance, TRIM21 promotes the ubiquitination and subsequent proteasome-dependent degradation of the M1 protein, thereby inhibiting the replication of H3, H5, and H9 subtypes of influenza A virus (IAV) [51]. Similarly, MARCH6 targets the viral NS5 protein of TMUV, leading to its TOLLIP mediated selective autophagic degradation and inhibition of viral replication [52]. In addition, certain ligases exert antiviral effects by positively regulating innate immune signaling. TRIM25 enhances the RIG-I-mediated signaling pathway, thereby suppressing DTMUV replication [53,54]. In response, avian viruses have evolved countermeasures. The V protein of NDV recruits the E3 ubiquitin ligase RNF5 to mediate MAVS polyubiquitination and degradation [55], and the H9N2 avian influenza PB1 protein exploits TRIM25 to induce K48-linked ubiquitination and autophagic degradation of MAVS [56]. In addition, the NS2B protein of TMUV recruits the E3 ubiquitin ligase MARCH5 to polyubiquitinate MAVS, thereby blocking the innate immune response and promoting viral replication [39]. However, most existing studies have focused on RNA-sensing pathways, and how avian DNA viruses manipulate the host ubiquitination system to regulate viral DNA-sensing pathways remains largely unexplored. Our study bridges this gap, in the present study, DPV infection upregulated duck RNF34 expression at both mRNA and protein levels, thereby promoting DPV proliferation in DEF cells, suggesting that DPV exploits RNF34 to suppress innate immunity.

Previous studies have established that RNF34 inhibits MyD88-mediated NF-κB activation and MAVS-driven IFN signaling. A recent study in teleost fish further reveals a conserved function, showing that RNF34 promotes nervous necrosis virus (NNV) immune evasion by targeting TBK1 and IRF3 for ubiquitination and degradation, thereby enhancing NNV replication [57]. We found that the E3 ubiquitin ligase RNF34 is a negative regulator of the cGAS-STING pathway in duck cells, which promotes the ubiquitination and degradation of duck cGAS, STING, and IRF7 through multiple mechanisms, thereby suppressing innate immune response. Interestingly, this mechanism is relatively conserved in chicken RNF34, although the chicken ortholog appears to specifically target chicken IRF7 for degradation. The comparison of amino acid sequences revealed that duck and chicken RNF34 have a high degree of homology (95%), which supports their conserved mechanism of targeting IRF7 to inhibit the cGAS-STING pathway. However, the homology between duck and chicken cGAS is 86%, while that for STING is only 70%. We propose that such differences in key components of the signaling pathway may alter the cellular context and thus influence the functional specificity of RNF34 substrate recognition. This may explain why duck RNF34 appears to target multiple components, while chicken RNF34 exhibits a more restricted activity primarily towards IRF7. Notably, duck RNF34 shares 67.12% homology with its teleost counterpart. This sequence divergence may underlie mechanistic differences: while teleost RNF34 directly degrades TBK1, duck RNF34 might suppress TBK1 kinase activity or disrupt the formation of its functional complexes, a hypothesis that warrants experimental validation. Our findings demonstrate that duck RNF34 promotes K11-linked polyubiquitination of duck cGAS, K11-/K33-linked polyubiquitination of duck STING, and K11-/K48-linked polyubiquitination of duck IRF7. The observed variation in ubiquitin linkage types may be attributed to the distinct three-dimensional structures and conformational features of duck cGAS, STING, and IRF7. These structural differences, particularly the local microenvironment surrounding the potential ubiquitination lysine residues, likely influence the topology of the polyubiquitin chains catalyzed by duck RNF34. In addition, ubiquitination is a multi-step enzymatic process involving E1, E2, and E3 enzymes, and different E2 enzymes exhibit intrinsic preferences for synthesizing specific types of ubiquitin chains [58]. It is therefore plausible that duck RNF34 may recruit distinct E2 conjugating enzymes to determine the resulting polyubiquitin chain topology, thereby fine-tuning the fate of each substrate. However, duck RNF34 mediates K11-linked polyubiquitination on all three substrates: duck cGAS, duck STING, and duck IRF7. Importantly, a growing body of evidence indicates that K11-linked ubiquitination plays versatile and crucial roles in protein degradation and pathway regulation, beyond being a rare modification. For instance: TRIM21 promotes K11-linked ubiquitination and proteasomal degradation of PHB2 [59]. TRIM29 negatively regulates antiviral signaling by inducing K11-linked ubiquitination and degradation of MAVS [60]. RNF167 mediates degradation of RIG-I/MDA5 via atypical K6/K11-linked ubiquitination [61]. These examples collectively demonstrate that K11-linked ubiquitination is a functionally significant signal employed by various E3 ligases, including TRIM and RING family members, to direct substrate degradation or modulation. Therefore, we speculate that duck RNF34 may possess an intrinsic catalytic preference for assembling K11-linked chains. This preference could be conferred through its specific interaction with E2 conjugating enzymes that favor K11-linkage formation.

Furthermore, duck RNF34 mediates the degradation of duck cGAS, STING, and IRF7 through multiple pathways, including the proteasome, autophagy, and apoptosis. This capacity to exploit several degradation mechanisms underscores the potent immunosuppressive function of duck RNF34 as a central negative regulator of innate immunity. The involvement of these multiple degradation pathways suggests that duck RNF34 may first initiate proteasomal tagging events, with misfolded/aggregated intermediates subsequently cleared by autophagy. Future studies are needed to determine the sequence of events and the relationship between duck RNF34 and autophagy receptors. While the roles of RNF34 in proteasomal and autophagic degradation have been previously reported [44,57], its involvement in modulating apoptosis, particularly in the context of antiviral immunity, warrants further investigation. One possibility is that duck RNF34 triggers specific caspase activation to dissociate immune signaling complexes. Elucidating how RNF34 influences apoptotic pathways to facilitate the turnover of immune signaling molecules could provide deeper insights into its comprehensive role in immune regulation. Collectively, these findings confirm that duck RNF34 is an evolutionarily conserved negative regulator of antiviral innate immunity.

The cGAS-STING signaling pathway serves as a fundamental component of innate immunity, critically involved in antiviral defense and inflammatory responses. Upon detection of cytosolic DNA, cGAS catalyzes the formation of cGAMP, which activates STING, leading to the assembly of the STING-TBK1-IRF3/7 signaling complex and ultimately inducing IFN-I production [62]. As a key downstream transcription factor, IRF3/7 must be phosphorylated and translocated into the nucleus to initiate this immune cascade. However, herpesviruses have evolved multiple strategies to target IRF3 and suppress IFN-I responses. For example, HSV-1 US3 kinase hyperphosphorylates IRF3 to inhibit IFN-β production, while VP24 impedes IRF3 activation [63,64]. PRV UL13 kinase phosphorylates IRF3 to promote its ubiquitin-mediated degradation [65]. These examples collectively illustrate that herpesvirus targeting of IRF3/7 represents a common immune evasion strategy. Notably, birds have undergone distinct adaptations in this pathway during evolution. The absence of an IRF3 homolog in avian species renders IRF7 indispensable for signal transduction and IFN induction in antiviral immunity [32,33]. Our previous research demonstrated that DPV infection suppresses immune responses in various duck primary cells [11]. In line with this, recent studies have reported multiple strategies employed by DPV to counteract host antiviral immunity. The DPV UL24 protein induces K48/K63-linked polyubiquitination of IRF7 to antagonize innate immunity [66]. The US3 kinase phosphorylates and promotes the degradation of both STING and IRF7 [21,22], while UL46 antagonizes innate immunity by activating the EGFR-PI3K-AKT pathway and disrupting the formation of the cGAS-STING-TBK1-IRF7 signalsome [23]. Nevertheless, the mechanisms by which DPV antagonizes innate immunity remain incompletely understood. In the present study, we applied the GST-pulldown coupled with mass spectrometry screening and Co-IP to identify LORF2 as viral proteins that interact with duck RNF34. We demonstrated that LORF2 recruits duck RNF34 to mediate the ubiquitination and degradation of duck IRF7, thereby inhibiting the duck cGAS-STING signaling pathway and promoting viral proliferation. Unlike proteins conserved across alphaherpesviruses, LORF2 is unique to DPV and lacks clear homologs in other well-characterized herpesviruses such as HSV-1, VZV, or PRV. This indicates that DPV has evolved a distinct genetic strategy to suppress host immunity via the ubiquitin system. LORF2 hijacks the duck host E3 ligase RNF34, which intrinsically exhibits broad substrate activity against multiple components of the pathway (cGAS, STING, and IRF7). By acting as a specificity factor, LORF2 effectively “reprograms” this host enzyme to selectively target the most critical component—IRF7—representing a highly efficient strategy tailored to exploit a key vulnerability in avian innate immunity. This selectivity may be attributed to LORF2 acting as a molecular adaptor to alter duck RNF34’s substrate specificity. Although duck RNF34 itself has broad affinity, its complex with LORF2 may undergo conformational changes that position duck IRF7 optimally for ubiquitination. Consistent with this model, during DPV infection, the degradation of duck IRF7, but not that of cGAS or STING, was directly dependent on duck RNF34. Moreover, knockdown of LORF2 restored IRF7 protein levels following DPV infection. Thus, we propose that DPV employs LORF2 to recruit duck RNF34, which subsequently mediates the ubiquitination and selective degradation of duck IRF7. This process effectively suppresses the host innate immune response and facilitates viral replication. This mechanism—targeted reprogramming of a host regulatory factor to maximize viral benefit—reflects a sophisticated level of hierarchical control in viral immune evasion. However, our co-immunoprecipitation results revealed a more complex interaction network: duck RNF34 can interact with duck cGAS, STING, TBK1, and IRF7, while LORF2 can interact with duck cGAS, STING, and IRF7. This suggests that the mechanisms by which LORF2 and duck RNF34 selectively target and regulate specific components of the signaling pathway under different physiological or pathological conditions remain to be fully elucidated. Future studies are needed to decipher the precise spatiotemporal regulation and functional outcomes of these interactions in detail.

In addition to LORF2, our AP-MS analysis also identified an interaction between duck RNF34 and the DPV protein US2. The US2 protein is highly conserved within the alphaherpesvirus subfamily [67]. Accumulating evidence strongly suggests that herpesviral US2 proteins are widely involved in modulating the host ubiquitin system and antiviral immune responses. For example, PRV US2 protein has been reported to interact with the host kinase ERK, regulating its subcellular localization [68]. A PRV US2-deletion mutant induced higher IFN-α secretion from plasmacytoid dendritic cells, indicating its potential role in suppressing antiviral immunity [69]. More importantly, a recent study demonstrated that PRV US2 recruits the host E3 ubiquitin ligase TRIM21 to catalyze K48-linked ubiquitination and degradation of STING, thereby inhibiting the cGAS-STING signaling pathway [70]. The HSV-2-encoded homolog US2 functions as a ubiquitin-interacting protein, directly engaging the ubiquitin system [71]. In DPV, US2 has been shown to be involved in viral penetration and cell-to-cell spread in vitro [72]. Our data show that DPV US2 also inhibits the cGAS-STING pathway. Therefore, DPV US2 is likely to suppress the immune response by utilizing the host ubiquitin mechanism through duck RNF34 or other factors. Whether DPV US2 cooperates with LORF2 in a coordinated viral strategy or targets distinct components of the signaling cascade presents a fascinating question for future research.

In avian herpesviruses, there are several genes that are unique only to avian herpesviruses, such as LORFs and SORFs, and these specific viral proteins play a key role in the viral life cycle and pathogenicity. Previous reports have indicated that the deletion of the MDV LORF2 homolog (vLIP) attenuates viral virulence [14]. Our earlier work also showed that the DPV-specific protein LORF3 facilitates viral replication both in vitro and in vivo, contributing to enhanced morbidity and mortality in infected ducks [17]. Similarly, LORF4 supports viral invasion and DNA replication [18], while LORF5 promotes DPV proliferation by enabling efficient cell-to-cell spread [19]. In this study, we establish that LORF2 acts as an immunosuppressive factor that antagonizes host antiviral immunity. Despite numerous attempts using a bacterial artificial chromosome (BAC) system harboring the cloned DPV genome, we were unable to generate a clean LORF2-knockout virus. This technical hurdle may stem from the possibility that LORF2 is essential for DPV replication under our experimental conditions. Additionally, the use of conditional knockdown technologies like CRISPRi could serve as a powerful future direction to definitively establish LORF2’s role in vivo. Importantly, our in vitro data show that siRNA-mediated knockdown of LORF2 causes a severe replication defect in DPV. This pronounced phenotype strongly suggests that LORF2 is indeed indispensable for efficient viral replication, offering a compelling biological explanation for the repeated failure to obtain a viable LORF2-knockout virus via recombination-based methods. Consequently, LORF2 emerges as a pivotal virulence factor, positioning it as an attractive potential target for developing attenuated live vaccines or antiviral therapies. Further investigations using animal models are warranted to clarify the precise impact of LORF2 on DPV pathogenicity and to dissect the underlying molecular mechanisms.

In summary, this study identifies the duck host E3 ubiquitin ligase RNF34 as a negative regulator of the duck cGAS-STING pathway and demonstrated its relatively conserved function in avian species. We further elucidate that the DPV-specific protein LORF2 recruits duck RNF34 to promote the ubiquitination and degradation of the key transcription factor duck IRF7, thereby suppressing the host antiviral immune response and facilitating viral proliferation. These results not only uncover a novel immune evasion strategy in which DPV utilizes specific viral protein to antagonize innate immunity, but also highlight the evolutionary arms race between viruses and their hosts. The conserved role of RNF34 across avian and fish species suggests that its exploitation represents a common viral mechanism for immune suppression. Our work thus provides a mechanistic foundation for understanding DPV pathogenesis and opens new avenues for developing broad-spectrum antiviral strategies that target virus-host ubiquitination networks.

## Supporting information

S1 Fig ChRNF34 targets IRF7 degradation and inhibits innate immunity. (A, B) CEF cells were co-transfected with chicken pIFNβ-Luc (A) or chicken pISRE-Luc (B) together with pRL-TK and ChRNF34 expression plasmids for 24 h, stimulated with poly(dA:dT) for 12 h, and then harvested for luciferase assay. (C-F) CEF cells were co-transfected with ChRNF34-Myc and ChcGAS-Flag (C), ChSTING-Flag (D), ChTBK1-Flag (E), or ChRF7-Flag (F) for 36 h. WCLs were analyzed by immunoblotting. (G) CEF cells were co-transfected with ChRNF34-Myc and ChIRF7-Flag for 24 h and treated with inhibitors for 12 h, followed by western blot analysis. (H) 293T cells were co-transfected with ChRNF34-Myc and ChIRF7-Flag plasmids for 36 h. WCLs were subjected to Co-IP with anti-Flag antibody and immunoblotting with indicated antibodies. (I) 293T cells were co-transfected with ChIRF7-Flag and ChRNF34-Myc plasmids for 36 h. WCLs were subjected to Co-IP with anti-Myc antibody and immunoblotting with indicated antibodies. (J, K) CEF cells were co-transfected with chicken pIFNβ-Luc (J) or chicken pISRE-Luc (K) together with pRL-TK, ChRNF34, and ChIRF7 expression plasmids for 36 h, and then harvested for luciferase assay. (L-O) CEF cells were transfected with ChIRF7 together with or without ChRNF34 expression plasmids for 36 h. chicken IFNβ (L), chicken MX (M), chicken OASL (N), and chicken PKR (O) mRNA levels were quantified by qPCR. Data are presented as mean ± standard error of the mean (SEM) and are representative of three independent experiments. Statistical significance was evaluated using two-tailed unpaired Student’s t-test. Ns, no significance; *, *P* < 0.05; **, *P* < 0.01; ***, *P* < 0.001; ****, *P* < 0.0001.
(TIF)

S2 Fig DuRNF34 interacts with US2 and LORF2. (A) DuRNF34 protein fused to GST (DuRNF34-GST) was expressed using the prokaryotic vector pGEX-4T-1 in *E. coli* BL21 and then purified it using glutathione agarose, followed by SDS-PAGE electrophoresis and Coomatis brilliant blue stainin. (B) Affinity purification combined with mass spectrometry analysis identified potential DPV viral proteins interacting with DuRNF34. (C-I) 293T cells were co-transfected with DuRNF34 and DPV viral protein eukaryotic expression plasmids, including US2 (C), LORF2 (D), LORF4 (E), UL2 (F), UL17 (G), UL25 (H) and UL31 (I), for 36 h. WCLs were subjected to Co-IP and immunoblotting with indicated antibodies. (J) DEF cells were co-transfected with duck pIFNβ-Luc, pRL-TK, and US2, together with DucGAS + DuSTING, DuSTING, DuTBK1, or DuIRF7 expression plasmids for 36 h, and then harvested for luciferase assay.
(TIF)

S3 Fig DuRNF34 and LORF2 promote DuIRF7 degradation in DEH cells. DEH cells were co-transfected with DuIRF7-Myc, DuRNF34-Flag, and LORF2-HA expression plasmids for 36 h. WCLs were analyzed by immunoblotting.
(TIF)
