# Supplementary figures and images for: Duck plague virus LORF2 utilizes RNF34 to inhibit antiviral innate immunity by ubiquitination and degradation of IRF7
Source: PLoS Pathog. 2026 Apr 24;22(4):e1014174. doi: 10.1371/journal.ppat.1014174 (PMC13152213; doi:10.1371/journal.ppat.1014174)

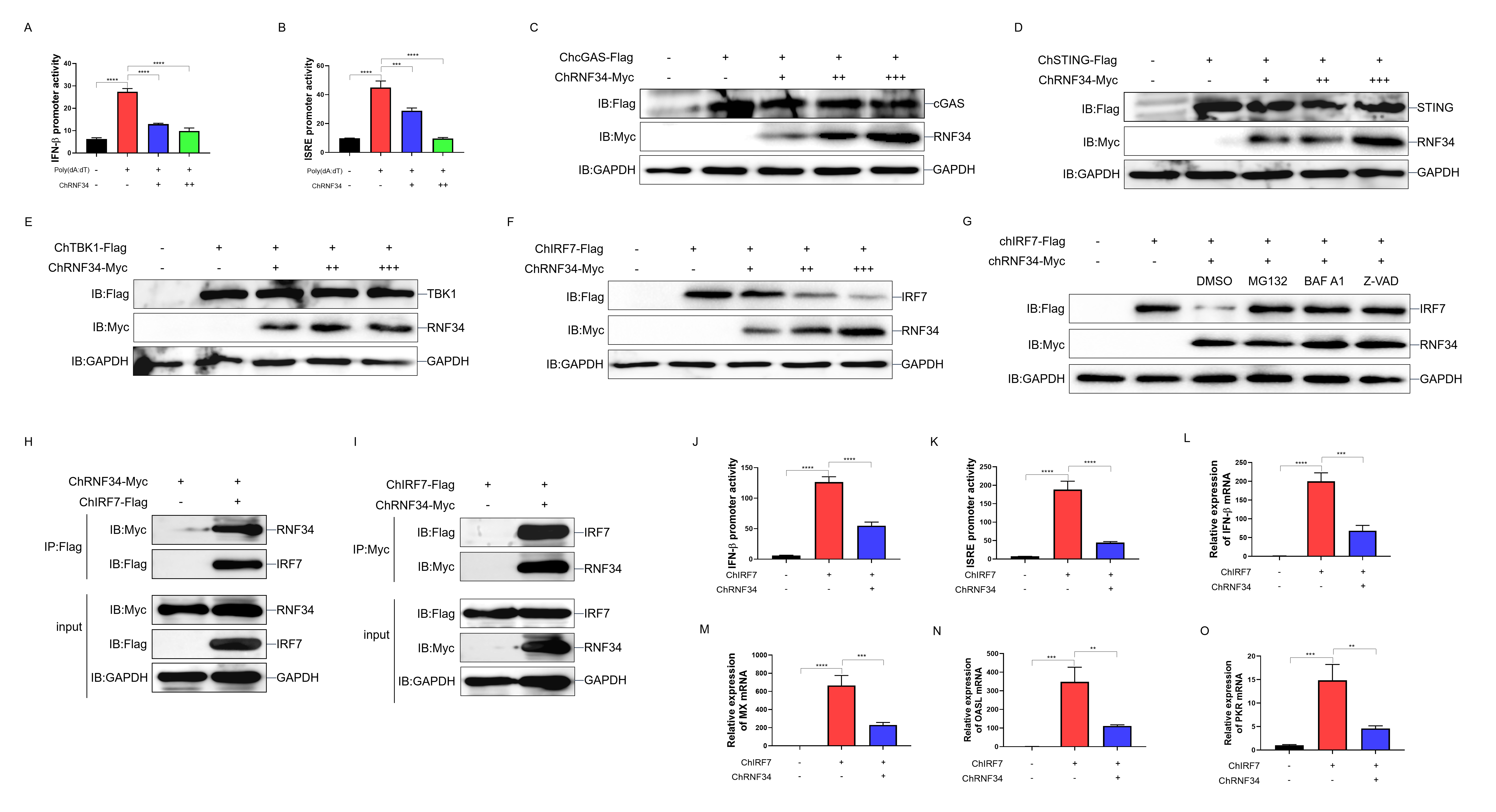

Supplement: S1 Fig — (A, B) CEF cells were co-transfected with chicken pIFNβ-Luc (A) or chicken pISRE-Luc (B) together with pRL-TK and ChRNF34 expression plasmids for 24 h, stimulated with poly(dA:dT) for 12 h, and then harvested for luciferase assay. (C-F) CEF cells were co-transfected with ChRNF34-Myc and ChcGAS-Flag (C), ChSTING-Flag (D), ChTBK1-Flag (E), or ChRF7-Flag (F) for 36 h. WCLs were analyzed by immunoblotting. (G) CEF cells were co-transfected with ChRNF34-Myc and ChIRF7-Flag for 24 h and treated with inhibitors for 12 h, followed by western blot analysis. (H) 293T cells were co-transfected with ChRNF34-Myc and ChIRF7-Flag plasmids for 36 h. WCLs were subjected to Co-IP with anti-Flag antibody and immunoblotting with indicated antibodies. (I) 293T cells were co-transfected with ChIRF7-Flag and ChRNF34-Myc plasmids for 36 h. WCLs were subjected to Co-IP with anti-Myc antibody and immunoblotting with indicated antibodies. (J, K) CEF cells were co-transfected with chicken pIFNβ-Luc (J) or chicken pISRE-Luc (K) together with pRL-TK, ChRNF34, and ChIRF7 expression plasmids for 36 h, and then harvested for luciferase assay. (L-O) CEF cells were transfected with ChIRF7 together with or without ChRNF34 expression plasmids for 36 h. chicken IFNβ (L), chicken MX (M), chicken OASL (N), and chicken PKR (O) mRNA levels were quantified by qPCR. Data are presented as mean ± standard error of the mean (SEM) and are representative of three independent experiments. Statistical significance was evaluated using two-tailed unpaired Student’s t-test. Ns, no significance; *, P < 0.05; **, P < 0.01; ***, P < 0.001; ****, P < 0.0001. (TIF) [file ppat.1014174.s001.tif]

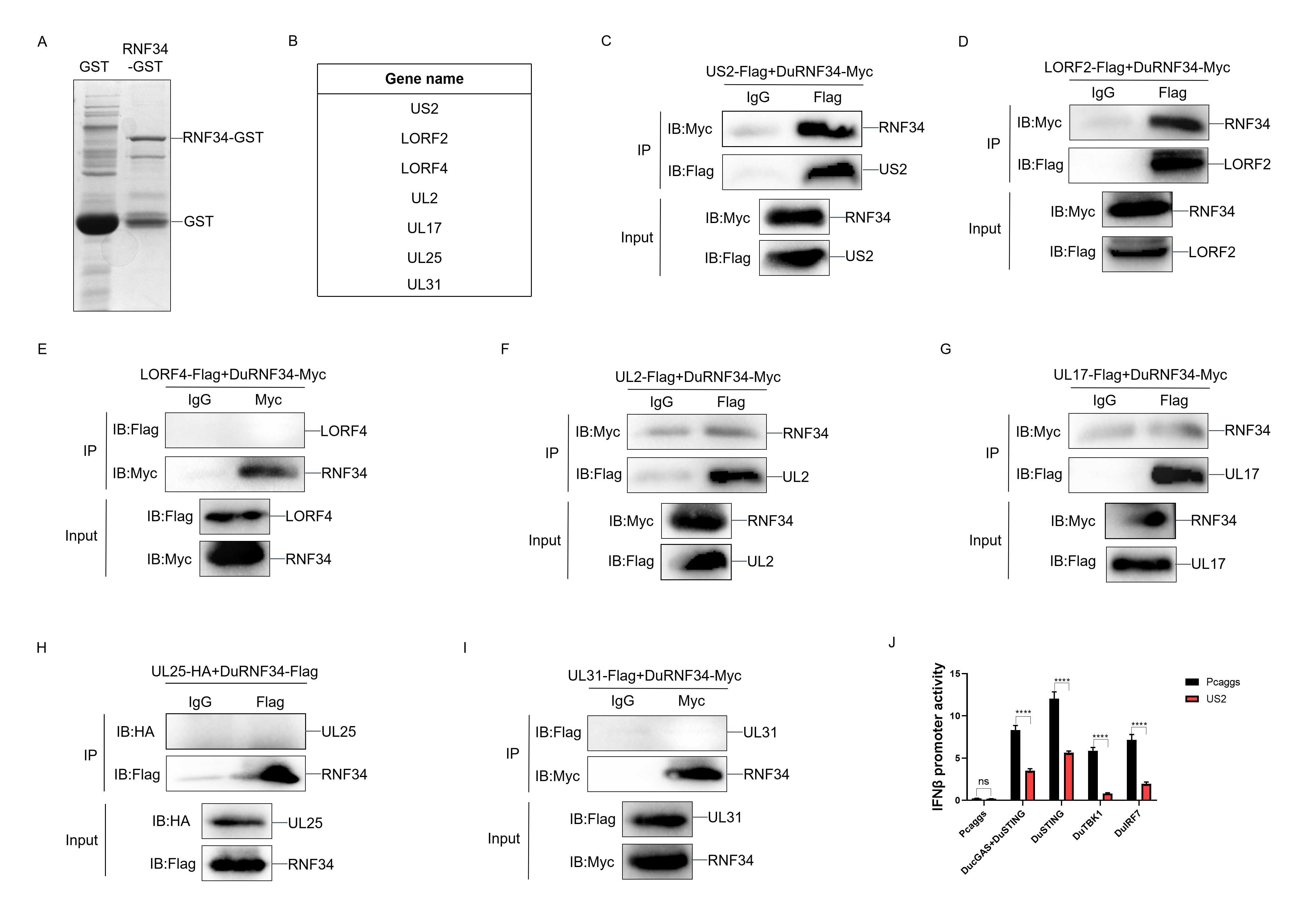

Supplement: S2 Fig — (A) DuRNF34 protein fused to GST (DuRNF34-GST) was expressed using the prokaryotic vector pGEX-4T-1 in E. coli BL21 and then purified it using glutathione agarose, followed by SDS-PAGE electrophoresis and Coomatis brilliant blue stainin. (B) Affinity purification combined with mass spectrometry analysis identified potential DPV viral proteins interacting with DuRNF34. (C-I) 293T cells were co-transfected with DuRNF34 and DPV viral protein eukaryotic expression plasmids, including US2 (C), LORF2 (D), LORF4 (E), UL2 (F), UL17 (G), UL25 (H) and UL31 (I), for 36 h. WCLs were subjected to Co-IP and immunoblotting with indicated antibodies. (J) DEF cells were co-transfected with duck pIFNβ-Luc, pRL-TK, and US2, together with DucGAS + DuSTING, DuSTING, DuTBK1, or DuIRF7 expression plasmids for 36 h, and then harvested for luciferase assay. (TIF) [file ppat.1014174.s002.tif]

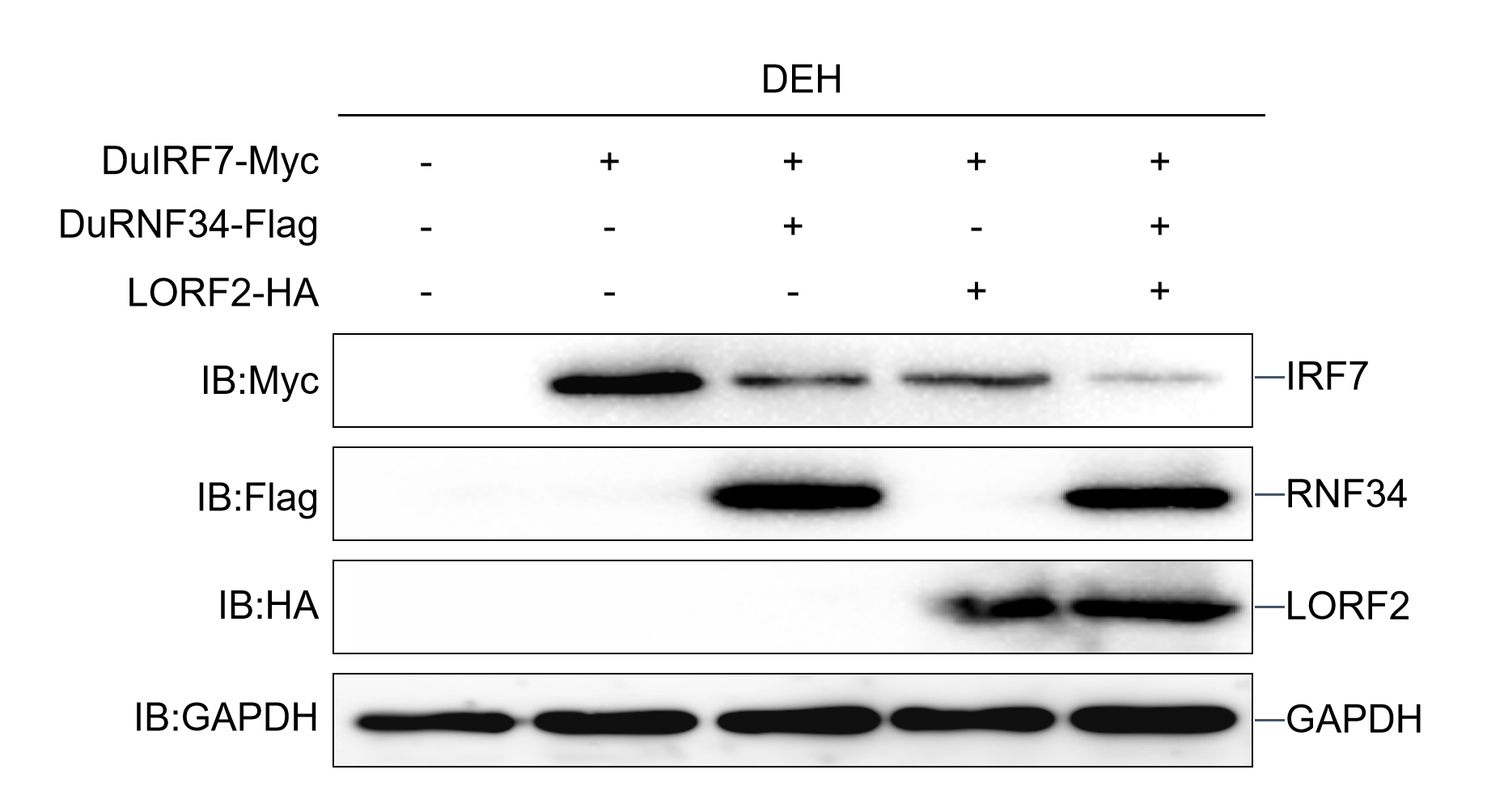

Supplement: S3 Fig — DEH cells were co-transfected with DuIRF7-Myc, DuRNF34-Flag, and LORF2-HA expression plasmids for 36 h. WCLs were analyzed by immunoblotting. (TIF) [file ppat.1014174.s003.tif]
